# Hepatocellular carcinoma cells remodel the pro-metastatic tumour microenvironment through recruitment and activation of fibroblasts via paracrine Egfl7 signaling

**DOI:** 10.1186/s12964-023-01200-6

**Published:** 2023-07-21

**Authors:** Bo Sun, Xiong Lei, Momo Cao, Yiming Li, Lian-Yue Yang

**Affiliations:** 1 Department of Hepatobiliary Surgery, Hunan Provincial People’s Hospital/The First Affiliated Hospital of Hunan Normal University, Changsha, 410005 Hunan China; 2 Liver Cancer Laboratory, Xiangya Hospital, Central South University, Xiangya Road 87, Changsha, 410008 Hunan China; 3 Department of General Surgery, Xiangya Hospital, Central South University, Changsha, 410008 Hunan China

**Keywords:** Hepatocellular carcinoma, Tumour microenvironment, Cancer-associated fibroblasts, EGF like domain multiple 7, Integrin α_ν_β_3_

## Abstract

**Background:**

The tumour microenvironment consists of a complex and dynamic milieu of cancer cells, including tumour-associated stromal cells (leukocytes, fibroblasts, vascular cells, etc.) and their extracellular products. During invasion and metastasis, cancer cells actively remodel the tumour microenvironment and alterations of microenvironment, particularly cancer-associated fibroblasts (CAFs), can promote tumour progression. However, the underlying mechanisms of the CAF formation and their metastasis-promoting functions remain unclear.

**Methods:**

Primary liver fibroblasts and CAFs were isolated and characterized. CAFs in clinical samples were identified by immunohistochemical staining and the clinical significance of CAFs was also analysed in two independent cohorts. A transwell coculture system was used to confirm the role of HCC cells in CAF recruitment and activation. qRT-PCR, western blotting and ELISA were used to screen paracrine cytokines. The role and mechanism of Egfl7 in CAFs were explored via an in vitro coculture system and an in vivo mouse orthotopic transplantation model.

**Results:**

We showed that CAFs in hepatocellular carcinoma (HCC) are characterized by the expression of α-SMA and that HCC cells can recruit liver fibroblasts (LFs) and activate them to promote their transformation into CAFs. High α-SMA expression, indicating high CAF infiltration, was correlated with malignant characteristics. It was also an independent risk factor for HCC survival and could predict a poor prognosis in HCC patients. Then, we demonstrated that EGF-like domain multiple 7 (Egfl7) was preferentially secreted by HCC cells, and exhibited high potential to recruit and activate LFs into the CAF phenotype. The ability of Egfl7 to modulate LFs relies upon increased phosphorylation of FAK and AKT via the receptor α_ν_β_3_ integrin. Strikingly, CAFs activated by paracrine Egfl7 could further remodel the tumour microenvironment by depositing fibrils and collagen and in turn facilitate HCC cell proliferation, invasion and metastasis.

**Conclusion:**

Our data highlighted a novel role of Egfl7 in remodelling the tumour microenvironment: it recruits LFs and activates them to promote their transformation into CAFs via the α_ν_β_3_ integrin signaling pathway, which further promotes HCC progression and contributes to poor clinical outcomes in HCC patients.

Video Abstract

**Supplementary Information:**

The online version contains supplementary material available at 10.1186/s12964-023-01200-6.

## Introduction

Hepatocellular carcinoma (HCC) is recognized as one of the most prevalent and lethal malignancies worldwide [1]. Liver resection remains the most important treatment choice. However, the clinical outcome for curative liver resection is still unfavourable due to the high rate of recurrence and metastasis [2, 3]. Meaningfully, our studies on HCC recurrence and metastasis for the past decade have found and defined a specific HCC subtype solitary large HCC (SLHCC) with a favorable prognosis similar to that of small HCC (SHCC),SLHCC also has significantly lower metastatic potential than nodular HCC (NHCC) [4]. Although the systematic studies on these three HCC subtypes have revealed some dominant oncogenes and tumour suppressor genes contributing to metastasis [5], more detailed mechanisms of HCC metastasis still need to be urgently explored from different perspectives.

The hallmarks of cancer indicate that cancer cells are not the only contributing factors in the manifestation of the disease [6]. Tumours are composed of cancer cells, extracellular matrix (ECM) and diverse stromal cells that include endothelial cells, inflammatory cells, bone marrow-derived myeloid cells, cancer-associated fibroblasts (CAFs), etc. [7]. These stromal components generate a favourable tumour microenvironment to support tumour growth, progression, and metastasis [8, 9]. The metastatic potential of cancer cells is determined by both their intrinsic properties and surrounding microenvironment properties. More importantly, during invasion and metastasis, cancer cells can actively remodel the tumour microenvironment, which in turn promotes tumour progression [10]. However, it is not yet well understood how cancer cells modulate the tumour microenvironment to promote tumour progression.

CAFs, the majority of stromal cells within the tumour microenvironment for many cancers, express distinctive biomarkers and mainly derive from quiescent resident fibroblasts [11]. Upon interaction with cancer cells, quiescent resident fibroblasts are recruited and activated, which promotes their transformation into CAFs [12]. CAFs and the complex set of signaling molecules they secrete generate an environment conducive to the desmoplastic stromal reaction, which in turn increases the invasiveness of the cancer cells [13]. In HCC, CAFs also frequently accumulate in the tumour microenvironment [13]. HCC cells can secrete lysophosphatidic acid and further recruit and activate resident fibroblasts, which promotes their transformation into CAFs [14]. CAFs can also affect the malignant phenotype of HCC cells by secreting cytokines and remodelling the extracellular matrix [15]. In spite of these studies, it is imperative to elucidate new critical molecular mechanisms affecting CAF formation and HCC progression, which will support the development of a new approach to HCC treatment.

In the present study, we aimed to determine how fibroblasts are recruited and activated to yield CAFs in HCC and further remodel the metastasis-promoting tumour microenvironment. We found that α-SMA could be a marker to represent CAF infiltration in HCC. High CAF infiltration in HCC tissues was associated with poor clinical characteristics and a poor prognosis. In addition, we further identified EGF-like domain multiple 7 (Egfl7) as a key tumour cell-secreted protein that promotes fibroblasts recruitment and activation. CAFs activated by Egfl7 through the α_ν_β_3_ integrin signaling pathway could further remodel the tumour microenvironment and in turn facilitate HCC cell proliferation, invasion and metastasis.

## Materials and methods

### Prognostic study

Two independent cohorts of subjects from two different centres were used for the prognostic study (Additional file 1: Fig. S1). The clinicopathologic characteristics of patients in the training cohort and validation cohort are shown in Additional file 2: Table S1. The diagnosis for all samples was confirmed by histopathological examination, and each sample had completed clinicopathologic data and follow-up data. In the training cohort (*n* = 176), paraffin-embedded HCC tissues were obtained from HCC patients undergoing surgical resection without any preoperative treatment at the Department of Surgery, the Xiangya Hospital of Central South University. In the validation cohort (*n* = 99), paraffin-embedded HCC tissues were obtained from HCC patients who underwent radical surgical resection without any preoperative treatment at the Department of Surgery, the Affiliated Cancer Hospital of Xiangya School of Medicine, Central South University. All HCC patients were regularly followed-up by the same experienced surgical team. The follow-up period was defined as the interval between the date of operation and that of the patient’s death, tumour recurrence or the last follow-up. Cases involving death from other causes were treated as censored cases. The follow-up status and any recurrence events were regularly updated for each patient.

### Cell lines

HepG2 and Hep3B cells were purchased from the American Type Culture Collection (ATCC, Rockville, MD). Bel-7402 and SMMC-7721 cells were gifted by the Tumour Institute of Central South University (Changsha, China). MHCC97-H and HCCLM3 cells were gifted from the Liver Cancer Institute of Fudan University (Shanghai, China). Short tandem repeat (STR) DNA fingerprinting was used to authenticate all cell lines before experiments. All cell lines were routinely cultured with the high glucose Dulbecco’s modified Eagle media (DMEM) (GIBCO, Gaithersburg, MD) supplemented with 10% foetal bovine serum (HyClone, Logan, UT), and maintained in 5% CO_2_ humidified incubator at 37 °C.

### Isolation and phenotypic characterization of primary fibroblasts

Samples of HCC and paired adjacent nontumourous liver tissue without cirrhosis were obtained from three patients undergoing liver resection. Approval for the study was obtained from the ethics committee and patients gave prior written informed consent for the use of their tissues. HCC and paired adjacent nontumourous liver tissues (ANLTs) were minced with scalpels in a tissue culture dish and then enzymatically dissociated in DMEM (GIBCO, Gaithersburg, MD) supplemented with 0.1% bovine serum albumin, 100,000 U/l penicillin G, 100 mg/l streptomycin, 1.0 g/ml fungizone, 500 units/mL collagenase D (Invitrogen, Carlsbad, CA), and 100 units/mL hyaluronidase (Zhongshan Goldenbridge Biotechnology, Beijing, China) at 37 °C for 16 h. The suspension was then centrifuged at 500 rpm for 5 min to separate the epithelial cells and fibroblasts. Fibroblasts in the supernatant were pelleted by centrifugation at 800 rpm for 10 min followed by two washes with DMEM medium. Fibroblast antigen–positive cells were isolated from the cell pellet by positive selection using anti-fibroblast MicroBeads and the MS Column (Miltenyi Biotec, Bergisch, Germany) according to the manufacturer’s instructions. Isolated cells were resuspended in DMEM supplemented with 15% FBS (HyClone) and plated in 25 cm^2^ tissue culture flasks. The cultures were then incubated at 37 °C in 5% CO_2_. Primary cultures of LFs and CAFs isolated from HCC were immunophenotypically characterized by positive immunostaining for the fibroblast marker vimentin and distinguished from tumour cells by the marker pan-cytokeratin, distinguished from endothelial cells by the marker CD31, and distinguished from haematopoietic cells by the marker CD45. Purification of the isolated fibroblast population was assessed by immunostaining. Cells from passages 3–10 were used for all experiments.

### Collagen gel-contraction assay

The collagen solution was poured into the wells and incubated for 1 h at 37 °C to allow the solution to polymerize. LFs and CAFs were layered on top of the formed lattices at a starting density of 1.5 × 10^5^ cells/cm^2^ and cultured in DMEM with 15% FBS for 48 h. Contraction of the lattices was monitored, and the lattice area was calculated. Gel diameter was measured at 24 and 48 h during culture. Contraction was calculated by measuring the area covered by the gel.

### Vector construction and transfection

The plasmids carrying short hairpin RNAs (shRNAs) for Egfl7 knockdown, the overexpression vector containing the Egfl7 ORF sequence, and corresponding controls were purchased from GeneChem Company (Shanghai, China). The sequences of shRNAs for Egfl7 knockdown and control shRNAs were reported in our previous study [16]. HCCLM3 cells were transfected with shRNA plasmids and SMMC-7721 cells were transfected with Egfl7 expression plasmids. Cells transfected with control plasmids were used as controls. Transfected cells were selected with 3 µg/mL puromycin. The efficiency of Egfl7 knockdown and overexpression was confirmed by qRT-PCR and western blotting.

### Quantitative real-time PCR (qRT-PCR)

Total RNA was extracted from HCC cell lines or fresh frozen tumour specimens by using TRIzol reagent (Invitrogen, Carlsbad, CA) according to the manufacturer’s instructions. qRT-PCR was performed using the SYBR® Green Real-time PCR Master Mix assay kit (Toyobo, Osaka, Japan) as described previously [17]. The primers for the corresponding genes are listed in Additional file 2: Table S2. The results were analysed using the 2^−ΔΔCt^ method.

### Western blot analysis

Total protein was separated by SDS-PAGE and then transferred onto PVDF membranes (Millipore, Bedford, MA). The blocked membranes were incubated with the primary antibodies followed by HRP-conjugated secondary antibodies (KPL, Gaithersburg, MD). Bands were visualized using an enhanced chemiluminescence kit (Santa Cruz Biotechnology, Santa Cruz, CA). The target signals were quantified by BandScan software (Bio-Rad, Hercules, CA) and defined as the ratio of target protein signal relative to β-actin signal. The antibodies used in the study are listed in Additional file 2: Table S3.

### Immunohistochemistry

Tumours specimens were formalin fixed prior to paraffin embedding. Sections were deparaffinized, rehydrated, and then subjected to antigen retrieval. The tissue sections were blocked with 1% BSA in Tris-buffered saline for 30 min prior to incubation with the primary antibody. Sections were then incubated with biotin-conjugated anti-rabbit/rat/goat IgG (Zhong-shan Golden-bridge Biotechnology, Beijing, China) for 20 to 30 min at room temperature. DAB was used as a detection system (Zhong-shan Goldenbridge Biotechnology) according to the manufacturer’s instructions. The following primary antibodies were used: mouse anti-α-SMA (Sigma, St Louis, MO), and rabbit anti-Egfl7 (Santa Cruz Biotechnology, Santa Cruz, CA). Negative control slides were probed with goat serum followed by the secondary antibody under the same conditions. The expression levels of Egfl7 were scored as described previously [16]. The staining of α-SMA was scored as follow: each tissue section was examined entirely for α-SMA staining intensity in interstitial fibroblasts. α-SMA-positive vessels of all sizes were easily distinguishable from interstitial fibroblasts. Overall, few α-SMA-positive vessels were detected in the tumours, and these were not included in the score. A scale of 0 to 3 was used (0 = no detected staining, 1 = weak staining, 2 = moderate staining, 3 = strong staining). Patients were then divided into two groups: a low α-SMA group (for scores of 0 and 1) and a high α-SMA group (for scores of 2 and 3).

### Cell proliferation and colony formation assays

For the cell proliferation assay, the cells were counted using a cell counter. For colony formation assays, equal cells were seeded into 35 mm dishes (Corning Costar Corp, Corning, NY) and cultured in 5% CO_2_ at 37 °C for 2 weeks. The number of colonies per dish was counted after staining with crystal violet. Only positive colonies (diameter > 40 μm) in the dishes were counted and compared. These experiments were performed in triplicate.

### Wound healing assay

HCC cells were seeded onto six-well culture plates (Corning Costar Corp) coated with fibronectin. After cells reached 100% confluence, to suppress cell proliferation which could confound the analysis of cell migration, cells were preincubated with mitomycin (Sigma, St. Louis, MO, 10 μg/ml) for 1 h at 37 °C. Wound healing assays were performed with a sterile pipette tip to make a scratch through the confluent monolayer. The medium was changed and the cells were cultured for 24 h. The percentage of wound closure was calculated for three randomly chosen fields.

### Transwell assay

For the transwell assay, approximately 1 × 10^5^ cells in serum-free medium were placed into the upper chamber of the insert with Matrigel (BD Biosciences, Franklin Lakes, NJ). After 24 h of incubation in 5% CO_2_ at 37 °C, the cells in the upper chamber were removed with cotton swabs, following fixed with 20% methanol, and then stained with a solution containing 0.1% crystal violet (Beyotime Biotechnology, Shanghai, China). The number of cells that adhered to the lower membrane of the inserts was counted. For each experimental group, the assays were performed in triplicate, and three random fields were chosen for analysis.

### Immunofluorescence

Cells were seeded in a 6-well culture plate (Corning Costar Corp) to prepare for immunofluorescence (IF). After incubation with primary antibodies, the cells were incubated with fluorescence-labeled secondary antibodies. The slides were photographed using the inverted fluorescence microscope a TE-2000S (Nikon, Tokyo, Japan). DAPI and fluorescence-labeled secondary antibodies were obtained from Beyotime Biotechnology (Shanghai, China).

### Enzyme-linked immunosorbent assay (ELISA) analysis

ELISAs were performed and analysed as described previously [18]. For the collection of conditioned medium (CM) from fibroblasts and HCC cells, cells were plated in 75cm^2^ flasks, washed twice with PBS 4 days later, and incubated for 48 h with serum-free DMEM. Then, the supernatant was harvested and centrifuged at 3,000 rpm for 5 min and was passed through a sterile Millipore 50 ml filtration system with a 0.45 mm PVDF membrane. The final CM was stored in a -80 °C refrigerator for later use.

Egfl7 levels were quantitatively measured by employing a sandwich ELISA system. First, a monoclonal antibody (Abnova, Taiwan, China) specific to Egfl7 was added to a 96-well microplate (Greiner Bio-One, Germany) as a capture antibody and incubated for 2 h at room temperature. The 96-well microplate was then coated at 4 °C overnight. The coated plate was washed in 0.05% phosphate-buffered saline/Tween (PBST). After washing away any unbound antibody, 4% bovine serum albumin (BSA) was added to the wells and incubated for 2 h at 4 °C for blocking. After washing with 0.05% PBST, 250-fold diluted serum samples were added to the wells and incubated for 2 h at room temperature. After washing away any unbound substances, a polyclonal antibody (Santa Cruz Biotechnology) specific for Egfl7 was added to the wells as a detection antibody and incubated for 2 h at room temperature. After removing any unbound antibody, goat anti-rabbit IgG antibody was added to the wells and incubated for 2 h. TMB soluble reagent (Tiangen Biotech, Beijing, China) was then added to the wells and allowed to react for 30 min at room temperature. The reaction was stopped by adding 1 M H_2_SO_4_ (Jingmei Biotech, Beijing, China). Colour intensity was determined by a photometer at a wavelength of 450 nm, with a reference wavelength of 570 nm. Recombinant Egfl7 protein (Abnova, Taiwan, China) was used as a standard sample in each assay.

### Xenograft orthotopic liver cancer model assays

To better simulate the microenvironment of tumour development in human HCC, a liver injection procedure was adopted. Male BALB/c nude mice aged 4–6 weeks were used. HCC cells alone or comixed with fibroblasts at a ratio of 1:4 within 0.1 ml Matrigel were injected into the left liver of each mouse. Six mice per group were used in all experiments. Only animals with accidental death were excluded from the data analysis. HCC cells were transduced with expression of control shRNA or Egfl7 shRNA, control vector or Egfl7 overexpression vector, and a vector encoding the red fluorescent protein (RFP) and a vector encoding firefly luciferase through plasmid infection. Animals were monitored by a bioluminescent imaging system (Xenogen, Hopkinton, MA). The metastasis-free survival time of the mice was recorded daily. The mice were sacrificed after 10 weeks, and all livers and lungs were harvested. The tumour size was calculated as follows: tumour volume (mm^3^) = (L × W^2^)/2, where L = long axis and W = short axis [19]. All livers and lungs were fixed with 10% phosphate-buffered neutral formalin, sectioned serially and stained with haematoxylin and eosin (H&E) or Masson’s trichrome for histological examination. Mice were performed and housed in the Animal Institute of CSU according to the protocols approved by the Medical Experimental Animal Care Commission.

### Statistical analysis

All data were analysed using the statistical software SPSS 18.0 for Windows (SPSS Inc., Chicago, IL). The differences between groups were analysed by Student’s* t* test between two groups or by one-way analysis of variance (ANOVA) in more than two groups when the variance was homogeneous. If the variance was not homogeneous, the differences between groups were analysed by the Mann–Whitney U test for two groups or by the Kruskal–Wallis H test for more than two groups. χ^2^ analysis was used to analyse the correlation between Egfl7 expression and clinicopathologic features. Survival curves were constructed using the Kaplan–Meier method and evaluated using the log-rank test. A Cox proportional hazards regression model was established to identify factors that were independently associated with the OS and DFS of HCC patients. All the tests were two-tailed and *P* < 0.05 was to indicate statistical significance.

## Results

### α-SMA as a marker of CAF infiltration in HCC

**Fig. 1:**
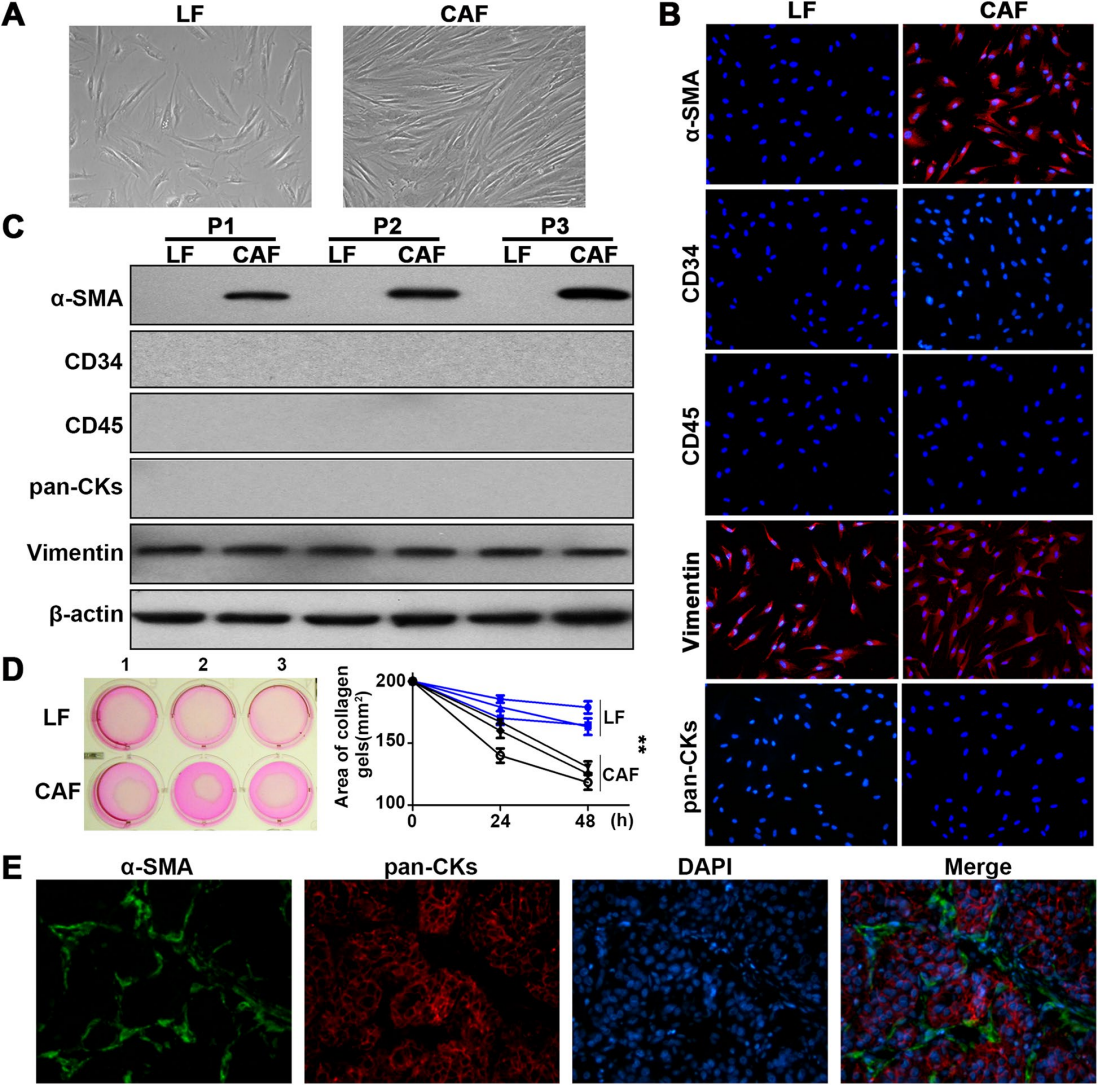
α-SMA as a marker of CAF infiltration in HCC. **A** Phase contrast microscopy images show the representative cellular morphology of liver fibroblasts (LFs) and cancer-associated fibroblasts (CAFs). **B**, **C** Immunofluorescence staining (**B**) and western blot (**C**) analysis of α-SMA, CD34, CD45 and vimentin in LFs and CAFs. **D** Collagen gel-contraction assay showed the collagen contraction ability of LFs and CAFs. **E** Dual immunofluorescence staining of pan-CKs and α-SMA in HCC tissues. **P < 0.01

We first isolated liver fibroblasts (LFs) and CAFs from adjacent nontumourous liver tissues (ANLTs) and HCC tissues respectively (Fig. 1A). Then, general markers for mesenchymal cells were detected in LFs and CAFs by IF and western blotting (WB). Both results showed that LFs and CAFs expressed vimentin, while they did not express the vascular marker CD34, myeloid marker CD45 or epithelial marker pan-cytokeratin (pan-CKs). Consistent with previous reports [20, 21], only CAFs expressed α-SMA, so this marker could be used to distinguish these two cell types (Fig. 1B-C). Further studies showed that CAFs had more powerful collagen contractility and proliferation abilities (Fig. 1D, Additional file 1: Fig. S2). In HCC tissues, dual immunofluorescence of pan-CKs and α-SMA showed α-SMA distributed between HCC cells, indicating that α-SMA expression could be used to represent CAF infiltration in HCC (Fig. 1E). These results indicate that we can distinguish CAFs from LFs based on both specific markers and biological behaviour.

### CAFs are frequently presented in HCC tissues and are associated with HCC metastatic potential

**Fig. 2:**
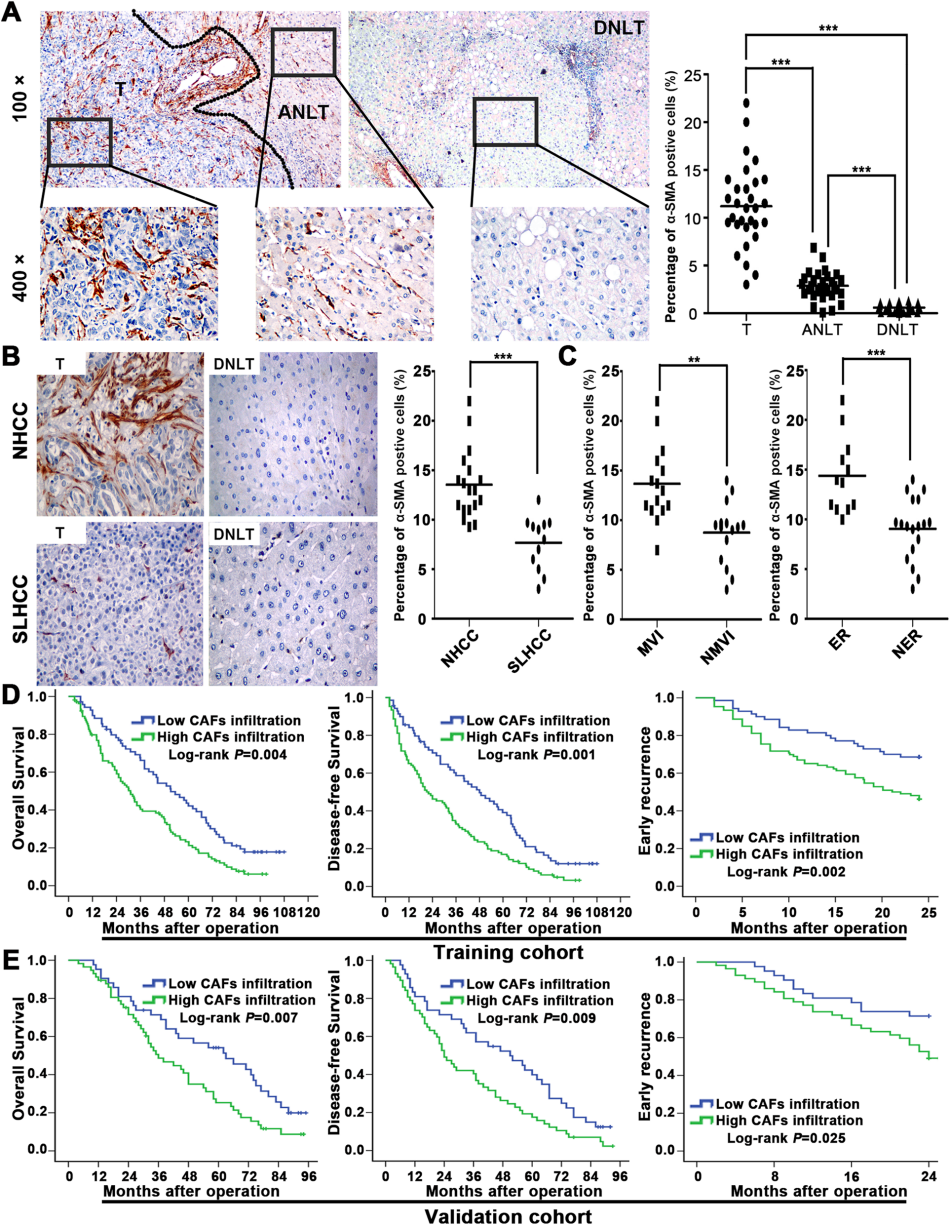
CAFs are frequently presented in HCC tissues and are associated with poor clinical characteristics and unfavourable prognosis in HCC patients. **A** Immunohistochemistry staining showed CAFs in HCC tissues, adjacent nontumourous liver tissues (ANLTs) and distant nontumourous liver tissues (DNLTs). **B** Immunohistochemistry staining showed CAFs in tumour tissues and DNLTs of nodular hepatocellular carcinoma (NHCC) and solitary large hepatocellular carcinoma (SLHCC). **C** Statistical analysis of CAFs in HCC tumour tissues with/without microvascular invasion (MVI) and with/without early recurrence (ER). **D**, **E** Kaplan–Meier analysis of overall survival, disease-free survival and early recurrence of HCC patients with high or low CAF infiltration in the training cohort (**D**) and validation cohort (**E**). **P < 0.01; ***P < 0.001

Based on the above results, we examined CAF infiltration in HCC by detecting α-SMA expression. Some interesting phenomena attracted our attention. First, immunohistochemistry (IHC) showed that CAFs were more abundant in HCC tissues than in ANLTs and CAF abundance was further increased in HCC tissues vs. distant nontumorous liver tissues (DNLTs), demonstrating a gradual decreasing trend (Fig. 2A). Second, NHCC with metastatic potential had more CAF infiltration than SLHCC (Fig. 2B). Third, CAF infiltration was also significantly higher in tumours with microvascular invasion (MVI) than in tumours without MVI. In addition, HCC tissues from patients with early tumour recurrences (within 2 years) also exhibited higher CAF infiltration than those from patients without early tumour recurrences (Fig. 2C). These results indicate CAFs have a certain role in promoting HCC progression.

### High CAF infiltration is associated with poor clinical characteristics and predicts a poor prognosis for HCC patients

As metastasis is the main causative factor for poor HCC outcome, we further determined the clinical value of CAFs in HCC according to REMARK guidelines for reporting prognostic biomarkers in cancer [22]. We first enrolled patients to set up training and validation cohorts and then classified patients in each cohort into high CAF infiltration and low CAF infiltration groups according to the α-SMA expression level (Additional file 1: Fig. S3A). In both cohorts, there were significantly more intratumoural CAFs than ANLTs. Intriguingly, NHCC had more CAF infiltration than SLHCC and SHCC, whereas the latter two with relatively favourable clinical outcomes had similar levels of CAF infiltration (Additional file 1: Fig. S3B). We next analysed the correlation between CAF infiltration and HCC clinicopathologic features. In the training cohort, high level CAF infiltration was positively correlated with tumour number, microvascular invasion, Edmondson-Steiner grade, TNM stage, and BCLC stage (*P* < 0.05) (Table 1). Similar results were further validated in the validation cohort (Table 1). Both univariable and multivariable analyses based on the Cox proportional hazard regression model both showed that in the training and validation cohort, CAF infiltration was an independent risk factor for both overall survival (OS) and disease-free survival (DFS) (*P* < 0.05) (Additional file 2: Tables S4,S5). Survival analysis of the training cohort further showed that high CAF infiltration in HCC tissues was associated with poor OS (*P* = 0.004) and DFS (*P* = 0.001). In addition, high CAF infiltration was associated with early tumour recurrence (*P* = 0.002) (Fig. 2D). Similar results were validated in another independent cohort (Fig. 2E). Above all, these results indicate that CAFs could predict a poor prognosis in HCC patients, which also suggests a supportive role of CAFs in HCC progression.

**Table 1:** Association between α-SMA expression and clinicopathologic variables of HCC patients in the training and validation cohort

**Clinicopathologic variables**	**No**	**Training cohort α-SMA expression levels**			**No**	**Validation cohort α-SMA expression levels**		
		**Low (*n* = 70)**	**High (*n* = 106)**	***P* value**		**Low (*n* = 42)**	**High (*n* = 57)**	***P* value**
**Gender**								
Female	30	11	19	0.703	24	11	13	0.698
Male	146	59	87		75	31	44	
**Age (years)**								
≤ 60	133	53	80	0.971	84	35	49	0.718
> 60	43	17	26		15	7	8	
**AFP (ng/mL)**								
≤ 20	65	24	41	0.554	31	14	17	0.710
> 20	111	46	65		68	28	40	
**HBsAg**								
Negative	54	20	34	0.622	23	11	12	0.550
Positive	122	50	72		76	31	45	
**Liver cirrhosis**								
Absence	73	27	46	0.525	43	18	25	0.921
Presence	103	43	60		56	24	32	
**Child–Pugh classification**								
A	155	59	96	0.208	89	38	51	0.870
B	21	11	10		10	4	6	
**Tumor number**								
Solitary	69	45	24	** < 0.001**	40	25	15	**0.001**
Multiple	107	25	82		59	17	42	
**Tumor size**								
≤ 5 cm	61	28	33	0.226	41	19	22	0.507
> 5 cm	115	42	73		58	23	35	
**Capsular formation**								
Absence	103	44	59	0.343	67	28	39	0.854
Presence	73	26	47		32	14	18	
**Microvascular invasion**								
Absence	76	39	37	**0.006**	39	22	17	**0.023**
Presence	100	31	69		60	20	40	
**Edmondson-Steiner grade**								
Low grade (I and II)	80	41	39	**0.005**	49	27	22	**0.012**
High grade (III and IV)	96	29	67		50	15	35	
**TNM Stage**								
I	55	30	25	**0.007**	37	26	11	** < 0.001**
II- III	121	40	81		62	16	46	
**BCLC Stage**								
0-A	52	32	20	** < 0.001**	34	21	13	**0.005**
B-C	124	38	86		65	21	44	

### Highly metastatic HCC cells can recruit and activate LFs to yield CAFs

**Fig. 3:**
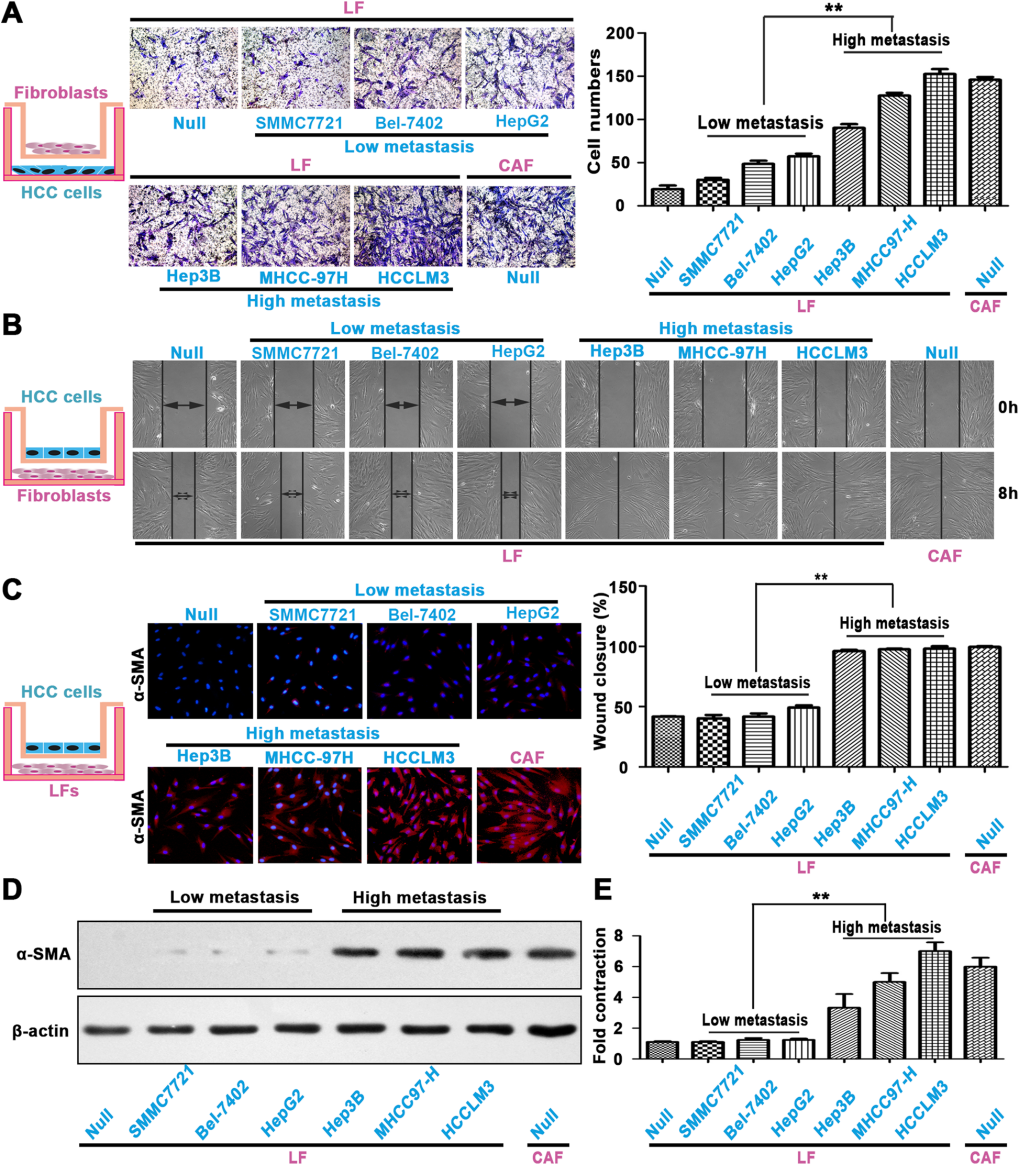
Highly metastatic HCC cells can recruit and activate LFs to yield CAFs. **A**, **B** Transwell migration assays (**A**) and wound-healing assays (**B**) were used to determine the role of highly metastatic HCC cell lines and less metastatic HCC cell lines in liver fibroblast (LF) migration. **C**, **D** Immunofluorescence staining (**C**) and western blotting analysis (**D**) of LFs using an α-SMA antibody after coculture with highly metastatic HCC cell lines and less metastatic HCC cell lines. **E** Collagen gel contraction assay confirmed LF contraction ability after treatment with conditioned medium from highly metastatic HCC cell lines and less metastatic HCC cell lines. **P < 0.01

Considering the important role of CAFs in HCC progression, we then asked where these CAFs in HCC come from. Based on the results shown in Fig. 2A, namely, a gradual decreasing trend of CAFs from HCC tissue to DNLT, we proposed that CAFs in HCC may be recruited and activated from adjacent LFs. To confirm this hypothesis, we first cocultured LFs with HCC cells, including the highly metastatic cell lines Hep3B, MHCC-97H, and HCCLM3 and the less metastatic cell lines SMMC-7721, Bel-7402, and HepG2, and then carried out a series of experiments. Transwell migration assays showed that highly metastatic cell lines, but not cell lines with low metastatic potential, significantly promoted LF migration (Fig. 3A). This phenomenon was also observed by wound-healing assays (Fig. 3B). LFs cocultured with highly metastatic cell lines, but not cell lines with low metastatic potential, exhibited α-SMA-positive phenotype, similar to the phenotype of CAFs (Fig. 3C). WB assays further confirmed that highly metastatic HCC cell lines induced LFs to adopt a CAF phenotype with high α-SMA expression (Fig. 3D). Collagen gel contraction assays further showed that LFs treated with conditioned medium from highly metastatic cell lines had higher contraction ability (Fig. 3E). These results confirm that highly metastatic HCC cell lines had the ability to promote LF migration and transformation into CAFs.

### Egfl7 is preferentially secreted by highly metastatic HCC cells and correlates with CAF infiltration in HCC

**Fig. 4:**
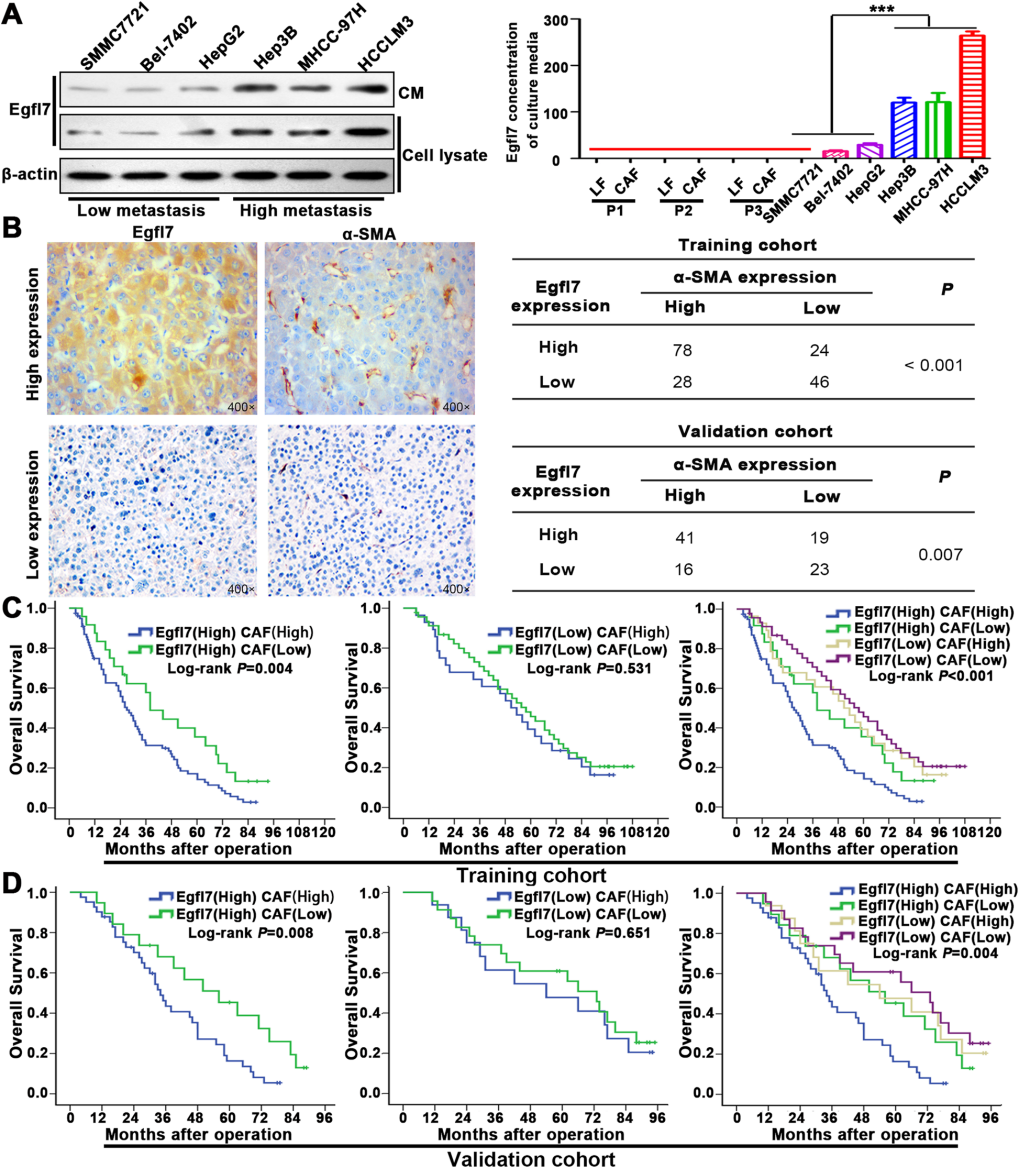
Egfl7 is preferentially secreted by highly metastatic HCC cells and correlates with CAF infiltration in HCC. **A** Egfl7 in HCC cell lysates and conditioned media from CAFs or LFs cocultured with the indicated HCC cells was detected by western blotting and ELISA. **B** Immunohistochemistry staining showed Egfl7 and α-SMA expression in serial sections of HCC tissues. The right tables show that Egfl7 was positively correlated with α-SMA in the training and validation cohorts. **C**, **D** Survival curves for overall survival in the training (**C**) and validation (**D**) cohorts after stratification based on Egfl7 expression and CAF infiltration. ***P < 0.001

The above coculture assays with LFs and HCC cells separated by transwell membranes indicated that CAF recruitment and activation may be driven by paracrine signaling. To screen the related factors, we first analysed eleven kinds of common tumour-secreted factors in HCC cells with high and low metastatic potential by quantitative PCR. The results showed that Egfl7 was the most preferentially expressed protein in highly metastatic HCC cells (Additional file 2: Table S6). WB and ELISA were further used to confirm that Egfl7 was secreted into conditioned media by highly metastatic HCC cells (Fig. 4A). However, we detected extremely low expression of Egfl7 in cell lysates and conditioned media from LFs or CAFs, indicating that Egfl7 is mainly derived from HCC cells (Fig. 4A; Additional file 1: Fig. S4A). We then asked whether Egfl7 was the crucial factor to recruit and activate LF in HCC. The Egfl7 expression level and its relationship with CAF infiltration in HCC were first analysed in public databases. Both HPA (https://www.proteinatlas.org/) and TIMER 2.0 (http://timer.comp-genomics.org/) databases showed that Egfl7 was significantly upregulated in HCC tissues compared with normal tissues (Additional file 1: Fig. S4B, S4C). Besides, in TIMER 2.0 database, we could also see Egfl7 was positively correlated with α-SMA in HCC (Additional file 1: Fig. S4D). Further analyses showed that Egfl7 was positively correlated with CAF infiltration from TIMER 2.0 EPIC, MCPCOUNTER, XCELL, TIDE algorithms in HCC (Additional file 1: Fig. S4E). IHC for serial sections of HCC tissues showed that Egfl7 expression was positively correlated with CAF infiltration (Fig. 4B). NHCC with high CAF infiltration had higher Egfl7 expression than SLHCC with low CAF infiltration (Additional file 1: Fig. S4F). In addition, Egfl7 expression demonstrated significant predictive value in survival-based stratification of patients with differential CAF infiltration in the training and validation cohorts (Fig. 4C-D). In the high Egfl7 expression groups, patients with high CAF infiltration had worse OS, whereas this trend was not observed in patients with low Egfl7 expression. In addition, those with high Egfl7 expression and high CAF infiltration had the poorest OS, whereas patients with low Egfl7 expression and low CAF infiltration had the most favourable OS. Above all, we identified an important secreted factor in HCC, Egfl7, and confirmed its close relationship with CAF infiltration.

### Egfl7 exhibits a potent ability to recruit and activate fibroblasts in HCC

**Fig. 5:**
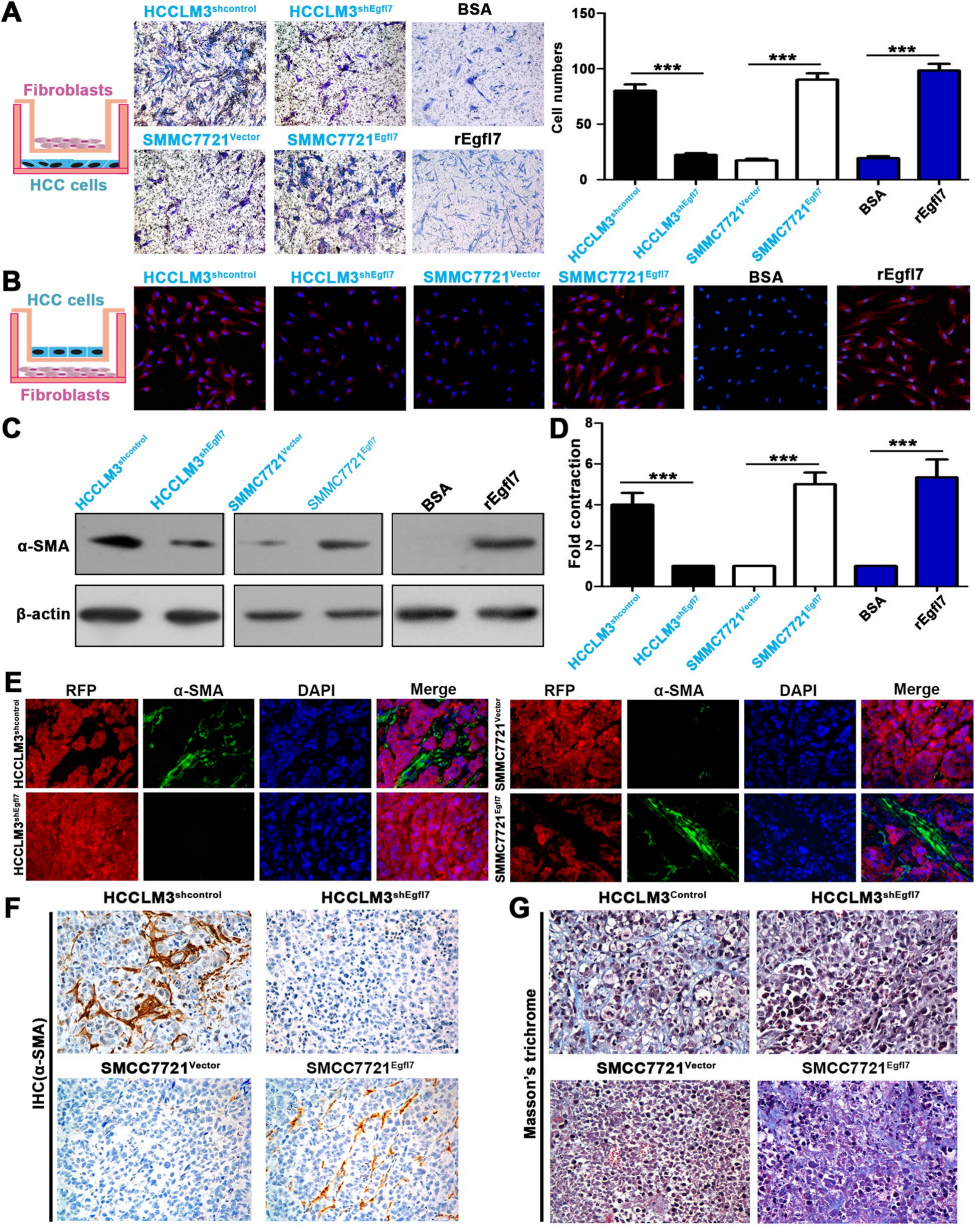
Egfl7 exhibits a potent ability to recruit and activate fibroblasts in HCC. **A** Transwell migration assays were used to detect the migratory ability of liver fibroblasts (LFs) after cocultured with HCCLM3^shEgfl7^, SMMC-7721^Egfl7^, or control cells or recombinant Egfl7 protein (rEgfl7). **B**, **C** Immunofluorescence staining and western blotting detected α-SMA expression in LFs after cocultured with HCCLM3^shEgfl7^, SMMC-7721^Egfl7^, their control cells or recombinant Egfl7 protein (rEgfl7). **D** Collagen gel contraction assays confirmed LF contraction ability after cocultured with indicated cells or rEgfl7. **E** Orthotopic tumours of mice were constructed using HCCLM3^shEgfl7^, SMMC-7721^Egfl7^ and their control cells with RFP gene expression. Dual immunofluorescence assay of orthotopic tumours showed red HCC cell and green CAF infiltration (α-SMA). **F** Immunohistochemistry staining showed α-SMA expression in orthotopic tumours from HCCLM3^shEgfl7^, SMMC-7721^Egfl7^ and their control cells. **G** Masson’s trichrome staining showed desmoplastic stroma in orthotopic tumours derived from HCCLM3^shEgfl7^, SMMC-7721^Egfl7^ and their control cells. BSA, bovine serum albumin; rEgfl7, recombinant Egfl7 protein (5 μg/mL). ***P < 0.001

Next, we assessed whether Egfl7 plays a role in recruiting and activating LFs to become CAFs in HCC. We selected a highly metastatic HCCLM3 cell line with high LF recruitment and activation ability and high Egfl7 expression and corresponding SMMC-7721 cell lines with opposite characteristics including low metastatic potential. We first constructed Egfl7 knockdown HCCLM3 cells and Egfl7-overexpressing SMMC-7721 cells. The efficiency of knockdown and overexpression was tested (Additional file 1: Fig. S5A; B). Then, we evaluated whether Egfl7 was responsible for LF recruitment and activation by in vitro coculture assays. Transwell migration assays showed that HCCLM3^shEgfl7^ cells had a lower ability to attract LF cells than HCCLM3^shcontrol^ cells, whereas SMMC-7721^Egfl7^ cells exhibited a markedly higher ability to attract LF cells than SMMC-7721^Vector^ cells (Fig. 5A). LF cells cocultured with HCCLM3^shcontrol^ cells, but not HCCLM3^shEgfl7^ cells, exhibited high levels of α-SMA expression and strong contractility, a typical phenotype of activated CAFs (Fig. 5B-D). Similarly, these hallmarks of CAF conversion were observed when LFs were cocultured with SMMC-7721^Egfl7^ cells but not SMMC-7721^Vector^ cells (Fig. 5B-D). Moreover, treatment with recombinant Egfl7 proteins significantly promoted LF migration (Fig. 5A) and mediated CAF conversion, as determined by detecting α-SMA expression (Fig. 5B-C) and fibroblast contractility (Fig. 5D). To further confirm the above results in vivo in an orthotopic tumour model, we transfected two pairs of HCC cell lines with the RFP gene, thereby allowing us to distinguish tumour cells and CAFs in HCC tumours (Additional file 1: Fig. S5C). Dual immunofluorescence assays showed that HCCLM3^shcontrol^ cell-derived tumours had more CAF infiltration than those derived from HCCLM3^shEgfl7^ cells. Similarly, more CAFs were presented in SMMC-7721^Egfl7^-derived tumours than in SMMC-7721^Vector^-derived tumours (Fig. 5E). Some studies have demonstrated that Egfl7 can promote tumour EMT and that HCC cells with EMT can also express α-SMA [23, 24]. The lack of overlap of RFP and α-SMA expression suggested that infiltrated CAFs were recruited but not generated via HCC EMT. The IHC assay also confirmed this result (Fig. 5F). In addition, Masson’s trichrome staining showed that HCCLM3^shcontrol^- and SMMC-7721^Egfl7^-derived tumours had more desmoplastic stroma with rich fibrils and collagen, indicating that CAFs infiltrating HCC tumours had active secretion activity (Fig. 5G). Therefore, these in vitro and in vivo results verified that HCC cell-secreted Egfl7 can recruit and activate LFs to become active CAFs.

### Egfl7 promotes CAF recruitment and activation through α_ν_β_3_ integrin signaling

**Fig. 6:**
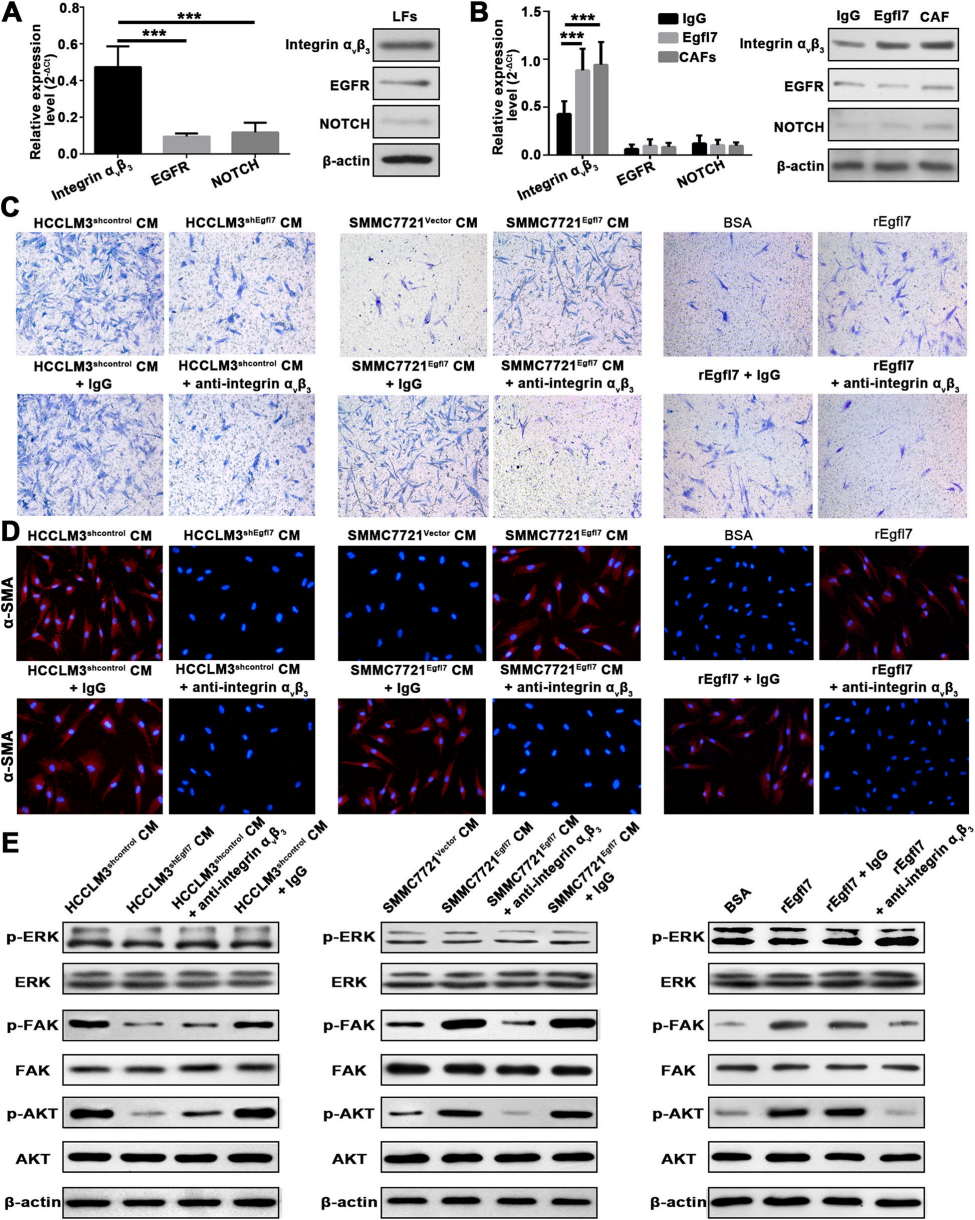
Egfl7 promotes CAF recruitment and activation through α_ν_β_3_ integrin signaling. **A** qRT-PCR and western blotting showed integrin α_ν_β_3_, EGFR and NOTCH expression levels in liver fibroblasts (LFs). **B** qRT-PCR and western blotting showed integrin α_ν_β_3,_ EGFR and NOTCH expression levels in CAFs and LFs stimulated and activated by Egfl7. **C** Transwell migration assays were used to detect the migratory ability of liver fibroblasts (LFs) cocultured with HCCLM3^shEgfl7^, SMMC-7721^Egfl7^, or control cells or recombinant Egfl7 protein (rEgfl7) after adding or not adding α_ν_β_3_ integrin neutralizing antibody. **D** Immunofluorescence staining detected α-SMA expression in LFs cocultured with HCCLM3^shEgfl7^, SMMC-7721^Egfl7^, or control cells or recombinant Egfl7 protein (rEgfl7) after adding or not adding α_ν_β_3_ integrin-neutralizing antibody. **E** Western blotting was used to analyse the activation of FAK, ERK and AKT signaling in LFs cocultured with HCCLM3^shEgfl7^, SMMC-7721^Egfl7^, or control cells or recombinant Egfl7 protein (rEgfl7) after adding or not adding α_ν_β_3_ integrin-neutralizing antibody. BSA, bovine serum albumin; rEgfl7, recombinant Egfl7 protein (5 μg/mL). ***P < 0.001

Other studies have found that Egfl7 often exerts its function through three kinds of receptors, that is epidermal growth factor receptor (EGFR), Notch and α_ν_β_3_ integrin [25]. Therefore, we first detected whether LFs express these three kinds of receptors. Both qRT‒PCR and WB showed that LFs had higher levels of α_ν_β_3_ integrin than EGFR and NOTCH (Fig. 6A). In addition, α_ν_β_3_ integrin expression, but not EGFR and NOTCH expression, was elevated in CAFs after LF stimulation and activation by Egfl7 (Fig. 6B). These results indicated that Egfl7 may recruit and activate LFs through α_ν_β_3_ integrin in HCC. To verify this hypothesis, we used an α_ν_β_3_ integrin neutralizing antibody to block its function in LFs. Conditioned medium (CM) from HCCLM3 cells with Egfl7 knockdown could not promote LF migration, and similar results were observed in CM from HCCLM3 cells treated with an α_ν_β_3_ integrin-neutralizing antibody (Fig. 6C). Likewise, CM from SMMC-7721^Egfl7^ cells could promote LF migration and this effect was blocked by adding an α_ν_β_3_ integrin neutralizing antibody to the CM (Fig. 6C) The result was further verified by using recombinant Egfl7 protein (Fig. 6C). Similarly, by detecting α-SMA expression and fibroblast contractility, we also confirmed that LFs were activated to yield CAFs by Egfl7 through α_ν_β_3_ integrin (Fig. 6D,Additional file 1: Fig. S6). Next, the potential molecular mechanisms were further explored. It has been reported that α_ν_β_3_ integrin exerts its effects via FAK-, ERK- or AKT-dependent pathways [26]. Thus, we examined the expression of key proteins in the FAK, ERK and AKT Signaling pathways by WB. The results showed that the expression of p-FAK and p-AKT was markedly increased after stimulation with CM from HCCLM3^shcontrol^ or SMMC-7721^Egfl7^ cells or with recombinant Egfl7 proteins, whereas this effect was antagonized by the addition of an α_ν_β_3_ integrin-neutralizing antibody. However, the expression of FAK, AKT, ERK, and p-ERK was not obviously affected (Fig. 6E). Collectively, these findings indicate that Egfl7 mediates CAF recruitment and activation in HCC through α_ν_β_3_ integrin and further activates AKT/FAK signaling in LFs.

### Egfl7-activated CAFs promote HCC proliferation, invasion and metastasis

**Fig. 7:**
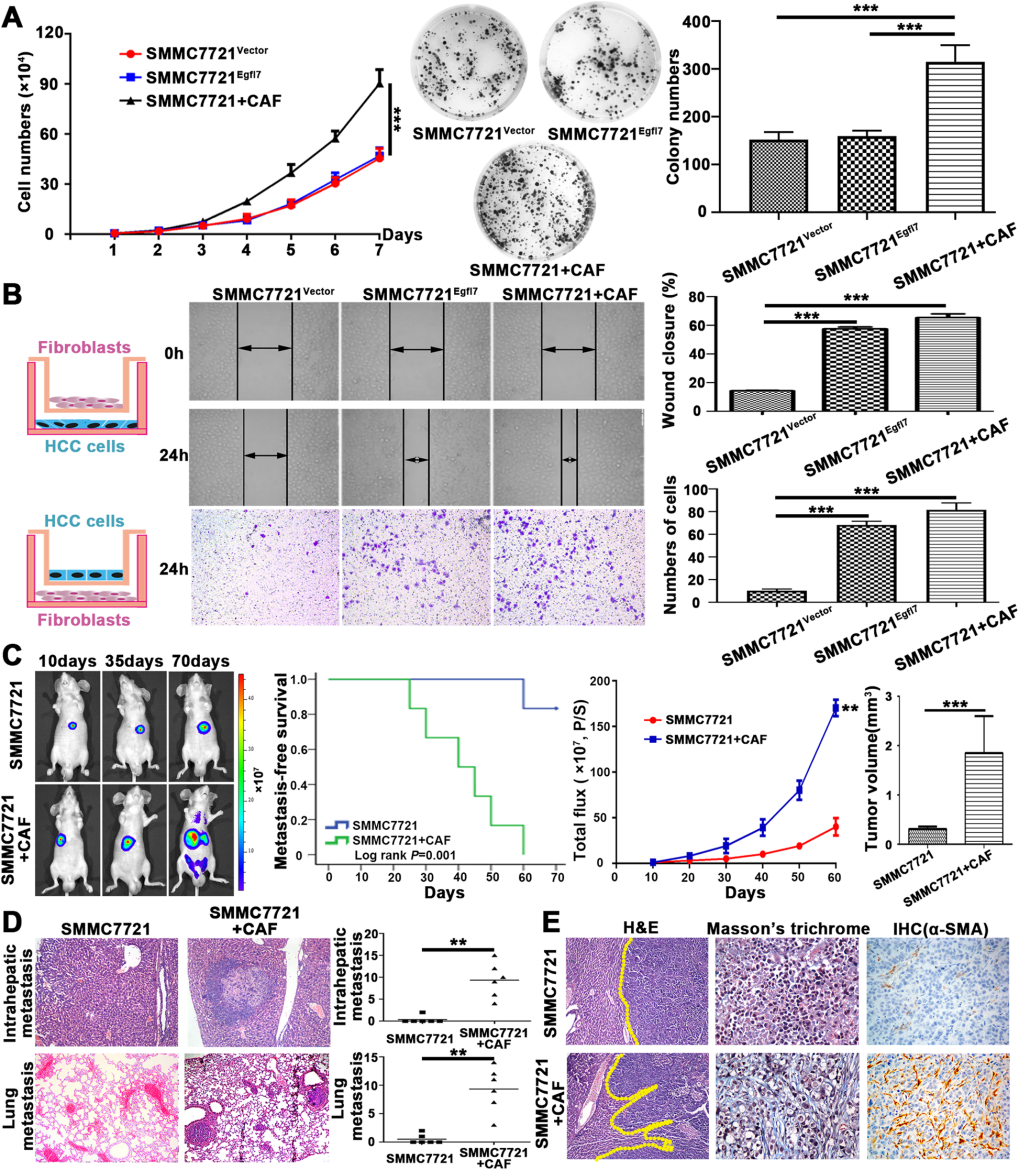
Egfl7-activated CAFs promote HCC proliferation, invasion and metastasis. **A** MTT and colony formation assays were used to determine the proliferative ability of SMMC-7721^Vector^, SMMC-7721^Egfl7^ and SMMC-7721 cells cocultured with CAFs. **B** Wound healing and transwell assays showed the migratory and invasive capacities of SMMC-7721^Vector^, SMMC-7721^Egfl7^ and SMMC-7721 cocultured with CAFs. **C** Orthotopic tumours of mice were constructed using SMMC-7721 and SMMC-7721 cells mixed with Egfl7-activated CAFs. The mouse survival and growth curves were depicted and final tumour volumes were compared. **D** The intrahepatic and lung metastasis numbers of orthotopic tumours from SMMC-7721 and SMMC-7721 cells mixed with Egfl7-activated CAFs were compared. **E** The tumour boundary, desmoplastic stroma and α-SMA expression in orthotopic tumours from SMMC-7721 and SMMC-7721 mixed with Egfl7-activated CAFs were determined by H&E, Masson’s trichrome and immunohistochemistry staining, respectively. ***P < 0.001

Our previous study revealed that Egfl7 had no effect on HCC cell proliferation in vitro but could significantly promote HCC growth in vivo [16]. In this study, we reconfirmed our previous results by in vitro colony formation assay, MTT assay and IVIS of an orthotopic tumour model (Additional file 1: Fig. S7). Considering the important role of Egfl7 in the HCC microenvironment, these contrary results prompted us to ask whether CAFs recruited and activated by Egfl7 could promote HCC proliferation, invasion and metastasis, thereby causing contradictory results in vitro and in vivo. Although overexpression of Egfl7 in SMMC-7721 cells caused no effect on their proliferation, coculture of SMMC-7721 cells with Egfl7-activated CAFs significantly promoted SMMC-7721 cell proliferation (Fig. 7A). In addition, CAFs could also promote SMMC-7721 cell migration and invasion (Fig. 7B). We then constructed orthotopic tumour model by SMMC-7721 cells mixed with or without CAFs. Tumours derived from SMMC-7721 mixed with CAFs grew more rapidly and metastasized more frequently than tumours derived from SMMC-7721 cells alone (Fig. 7C-E). The results in Fig. 5 showed that SMMC-7721 cells had a poor ability to recruit and activate LFs, as indicating by very low expression of α-SMA in tumours derived from SMMC-7721^vector^ cells. Thus, the effect of mouse derived LF on the tumours could be neglected and the tumour promoting effect mainly came from the mixed human CAFs. These results demonstrated that CAFs were activated by Egfl7 and could further promote HCC progression.

## Discussion

Increasing evidence has indicated that CAFs, as activated fibroblasts in the cancer stroma, are important mediators of tumour metastasis [27]. The better understanding of molecular mechanisms for fibroblast recruitment and activation and the tumour-promoting properties of CAFs in HCC are of great importance to understand HCC metastasis. CAFs, characterized by α-SMA expression, promote tumour progression by directly or indirectly stimulating tumour cell proliferation, and invasion, and by enhancing angiogenesis, which is the main cause of a poor prognosis in HCC patients [28, 29]. Here, in our study, we isolated two totally different kinds of fibroblasts in ANLTs and HCC tissues with significantly contrasting expression phenotypes and biological functions. CAFs had a more powerful ability of collagen contractility and proliferation. In addition, CAFs of HCC could be easily distinguished from LFs by detecting α-SMA, which is consistent with previous studies [20]. Although many studies have demonstrated that CAFs can promote HCC progression [21, 30], their clinical significance in HCC has been less explored. Our results showed that high CAF infiltration was significantly related to almost all previously known malignant clinicopathological variables, such as tumour number, tumour differentiation, vascular invasion, pathologic satellite nodules, and TNM stage. Of note, patients with high CAF infiltration presented more poorly differentiated cancers than those with low CAF infiltration, suggesting that stromal content likely had a direct interaction with tumour differentiation during the natural history of HCC. In addition, CAF infiltration in HCC was an independent risk factor for both OS and DFS. Moreover, survival analysis indicated that HCC patients with high CAF infiltration showed poor DFS and OS. Through systematic analysis, we confirmed the clinical significance of CAFs in HCC, which indicated that CAFs may play an important role in HCC progression.

CAFs are a heterogeneous population of cells in tumour and their source is not clearly defined. Our IHC results showed a gradual decreasing trend of CAFs from HCC tissues to DNLTs, which suggests that CAFs in HCC tissues may come from adjacent LFs. Indeed, we confirmed that highly metastatic HCC cells could recruit and activate LFs to yield CAFs by coculture assays. In addition, we screened the proteins secreted by highly metastatic HCC cells for CAF recruitment and activation and found that Egfl7 was the most differentially expressed protein between HCC cells with high metastatic potential and those with low metastatic potential. We further confirmed that Egfl7 in HCC was significantly correlated with CAF infiltration. The combination of Egfl7 with CAF infiltration could provide more information for predicting HCC patient prognosis. Many previous studies have reported that Egfl7 can promote HCC progression [16, 31, 32]. However, these studies mainly focus on the role of Egfl7 in HCC cells. A recent study by our team has identified serum Egfl7 level as a valuable marker for the diagnosis of early HCC [18], suggesting HCC cells can secrete Egfl7 and may play a certain role in HCC tumour microenvironment. Egfl7 can also modulate tumour immune microenvironment by mediating immune cell infiltration [33]. Egfl7 overexpressing epidermal stem cells can promote fibroblast proliferation and migration [34]. However, the association between Egfl7 and CAF infiltration has not been reported in HCC yet. Here, our in vitro and in vivo assays both showed that CAF recruitment and activation occurred mainly through Egfl7 secreted by HCC cells but not caused by Egfl7-induced EMT. Therefore, in this study, we extended the knowledge of Egfl7 in HCC by showing that Egfl7 plays a paracrine role in remodelling the tumour microenvironment by recruiting and activating CAFs. By systematically investigating the role of Eglf7 on CAFs in HCC microenvironment, we could not only understand new mechanisms of HCC progression, but also provide new ideas for the treatment of HCC recurrence and metastasis.

In glioma, a continuous Egfl7 autocrine flow line was the cause of oncogenic activation of EGFR [35]. Other studies have shown that Egfl7 acts as an endogenous antagonist of Notch signaling that regulates the proliferation and differentiation of neural stem cells [36]. Egfl7 could also promote pituitary adenomas proliferation and invasion via the Notch2/DLL3 signaling pathway [37]. Huang C et al. demonstrated that Egfl7 could ligate α_v_β_3_ integrin to enhance vessel formation [38]. Considering the various functions of Egfl7, we further asked how Egfl7 recruits and activates CAFs in HCC. Intriguingly, we found that LFs express more α_v_β_3_ integrin than EGFR and NOTCH receptors and that only α_v_β_3_ integrin levels of LFs had increased after cocultured with Egfl7. We then further confirmed that Egfl7 could recruit and activate CAFs via α_v_β_3_ integrin. Intracellular Signaling was also tested and the results showed that AKT/FAK signaling was activated, followed by Egfl7 binding to α_v_β_3_ integrin. In this study, we clarified a new mechanism of CAF transformation in HCC involving αvβ3 integrin/FAK/Akt signaling.

Stromal CAFs are rich sources for growth factors, survival factors and proangiogenic factors in tumour formation and progression [39]. Moreover, CAFs remodel the tumour ECM with a significant increase in fibrils and collagen content [40]. Our study provided evidence that Egfl7- activated CAFs could further promote HCC progression. CAFs support the malignant phenotype of HCC by promoting growth, metastasis and ECM organization. Our previous study showed that Egfl7 had no effect on HCC cell proliferation in vitro but could significantly promote HCC growth in vivo [16]. Therefore, these results confirmed that tumour biological behaviours were determined not only by tumour cells themselves but also by their surrounding microenvironment. HCC cells could actively remodel the tumour microenvironment and further facilitate tumour progression. Therefore, our study elaborated on a vicious circle in which HCC cells recruit and activate CAFs through α_v_β_3_ integrin/FAK/Akt signaling by secreting Egfl7, and Egfl7-activated CAFs could in turn promote HCC progression.

## Conclusions

Our study showed that Egfl7 expression in HCC promotes tumour progression in a paracrine manner by recruiting and activating LFs into CAFs, which results from elevated FAK and AKT phosphorylation by signal transduction of α_ν_β_3_ integrin. From a therapeutic perspective, this study provides a rationale for clinical testing of the concept of therapeutic targeting of CAFs in HCC.

## Supplementary Information

**Additional file 1: Fig. S1.** Flow diagram for clinical study design with two independent cohorts of HCC patients enrolled. **Fig S2.** The proliferative ability of primary liver fibroblasts (LFs) and cancer-associated fibroblasts (CAFs). **Fig S3.** CAFs infiltration in HCC clinical samples. **Fig S4.** Egfl7 expression in LFs/CAFs and HCC clinical samples. **Fig S5.** Establishment of working cell lines. **Fig S6.** Collagen gel contraction assays were used to detect contraction ability of indicated liver fibroblasts (LFs). **Fig S7.** In vitro and in vivo assays to confirm the biological roles of Egfl7 in HCC cells.

**Additional file 2: Table S1.** Clinicopathologic variables of HCC patients in the training cohort and validation cohort. **Table S2.** The primers of gene used for qRT-PCR. **Table S3.** Antibodies used in this study. **Table S4.** The Cox proportional hazard regression analyses for disease-free survival (DFS) and overall survival (OS) in the training cohort. **Table S5.** The Cox proportional hazard regression analyses for disease-free survival (DFS) and overall survival (OS) in the validation cohort. **Table S6.** A list of the secreted proteins differentially expressed by HCC cells with different metastatic potential.

## Data Availability

The datasets used and/or analysed during the current study available from the corresponding author on reasonable request. All data generated or analysed during this study are included in this published article.

## Abbreviations

HCC: Hepatocellular carcinoma
SLHCC: Solitary large hepatocellular carcinoma
NHCC: Nodular hepatocellular carcinoma
SHCC: Small hepatocellular carcinoma
Egfl7: Epidermal growth factor-like domain 7
TNM classification: Tumour-node-metastasis classification
BCLC stage: Barcelona Clinic Liver Cancer stage
OS: Overall survival
DFS: Disease-free survival
qRT-PCR: Quantitative Real-time PCR
