# Supplementary material for: Hepatocellular carcinoma cells remodel the pro-metastatic tumour microenvironment through recruitment and activation of fibroblasts via paracrine Egfl7 signaling
Source: Cell Commun Signal. 2023 Jul 21;21:180. doi: 10.1186/s12964-023-01200-6 (PMC10362567; doi:10.1186/s12964-023-01200-6)
Supplement: Supplementary file 2 — Additional file 1: Fig. S1. Flow diagram for clinical study design with two independent cohorts of HCC patients enrolled. Fig S2. The proliferative ability of primary liver fibroblasts (LFs) and cancer-associated fibroblasts (CAFs). Fig S3. CAFs infiltration in HCC clinical samples. Fig S4. Egfl7 expression in LFs/CAFs and HCC clinical samples. Fig S5. Establishment of working cell lines. Fig S6. Collagen gel contraction assays were used to detect contraction ability of indicated liver fibroblasts (LFs). Fig S7. In vitro and in vivo assays to confirm the biological roles of Egfl7 in HCC cells. [file 12964_2023_1200_MOESM1_ESM.doc]

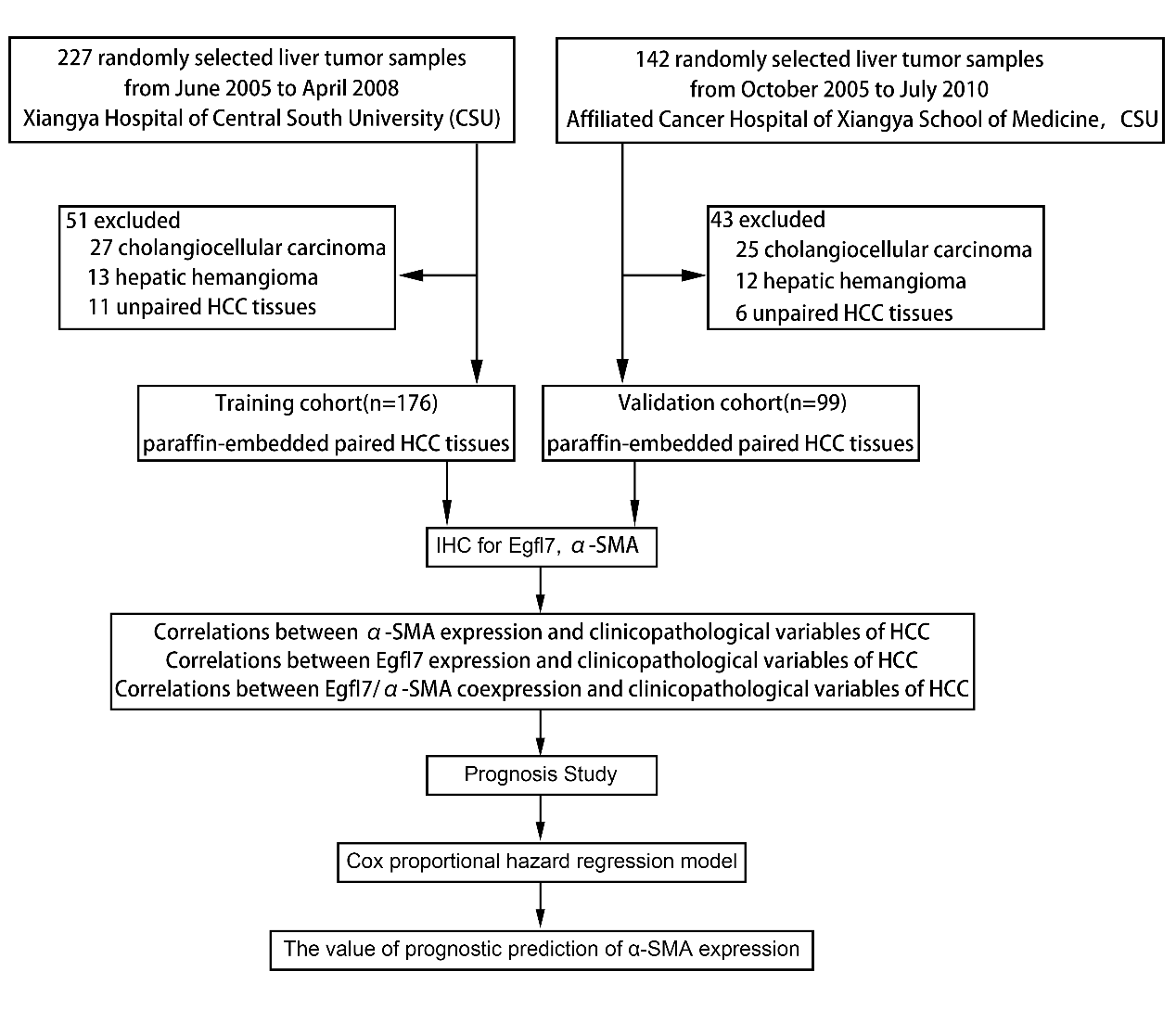


**Fig. S1. Flow diagram for clinical study design with two independent cohorts of HCC patients enrolled.**


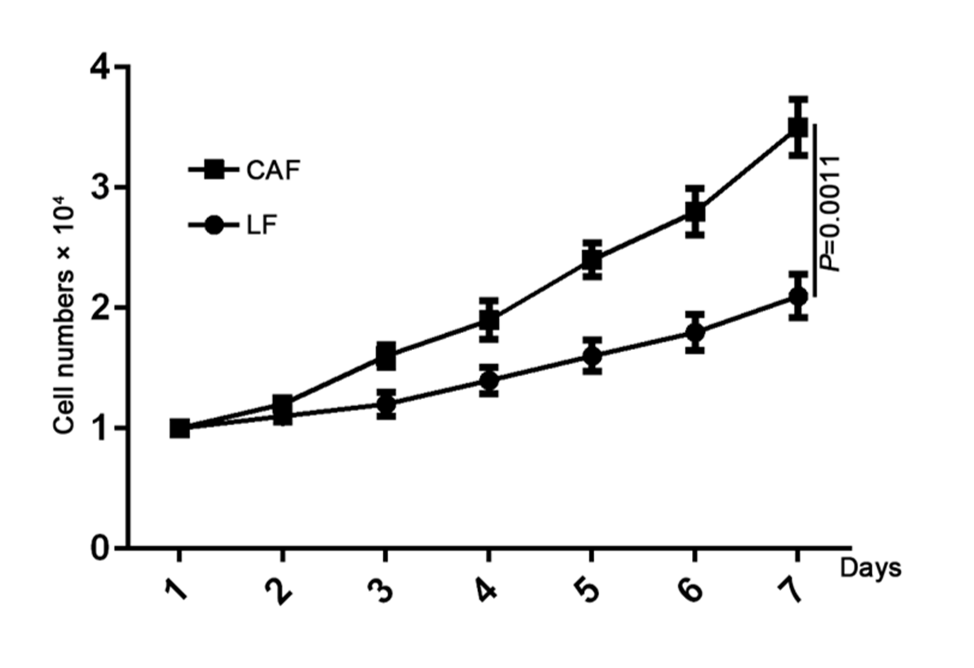


**Fig S2. The proliferative ability of primary liver fibroblasts (LFs) and cancer-associated fibroblasts (CAFs).** MTT assay was used to determine proliferation of LFs and CAFs.

**
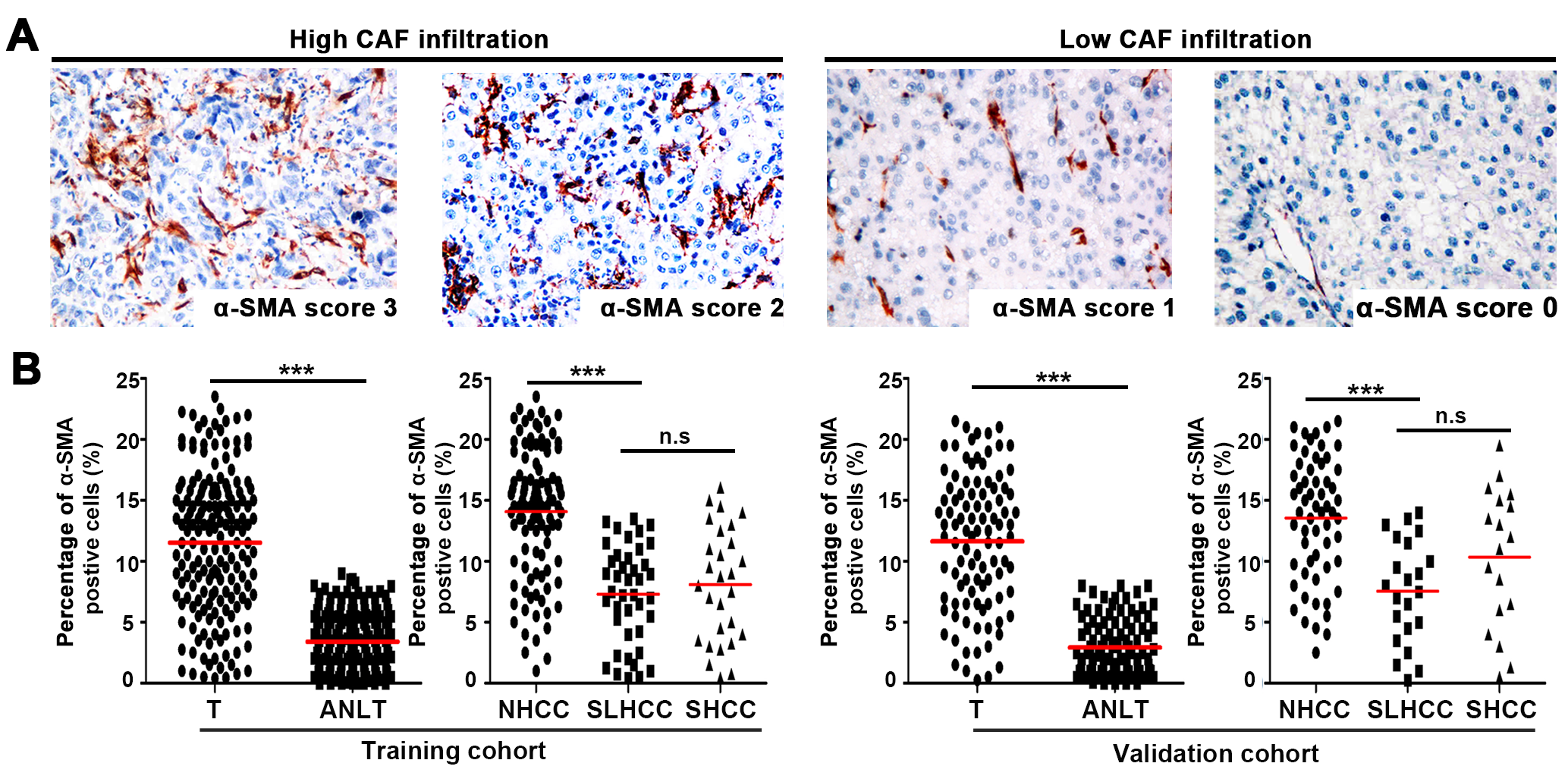
**

**Fig S3. CAFs infiltration in HCC clinical samples.** (A) Representative immunohistochemical images showed high CAF infiltration and low CAF infiltration according to α-SMA expression level. (B) The number of CAFs in HCC tumor tissues and adjacent nontumorous liver tissues (ANLTs), and different clinical subgroups from the training cohort and validation cohort were compared. n.s, no significance; ****P* < 0.001.

**
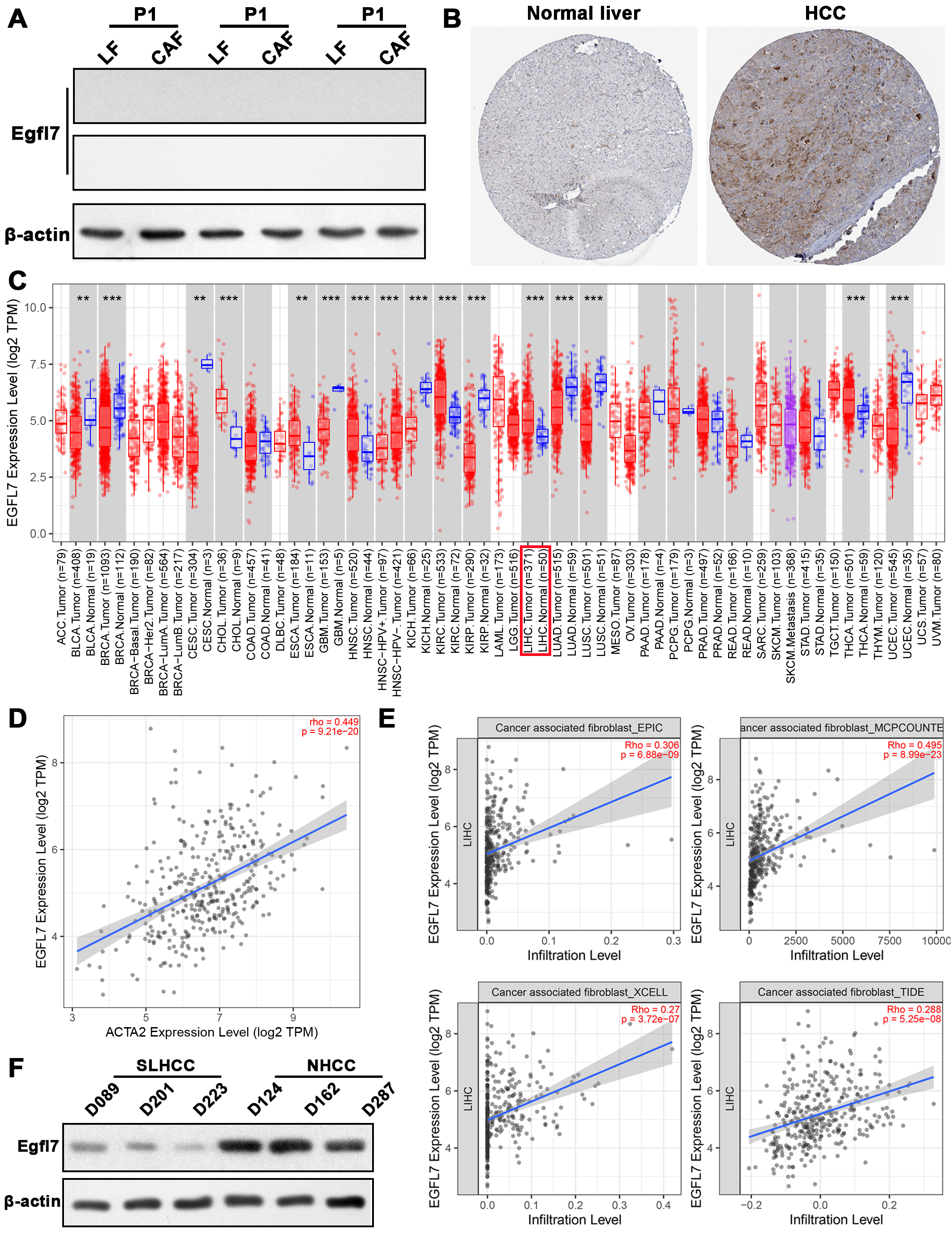
**

**Fig S4. Egfl7 expression in LFs/CAFs and HCC clinical samples.** (A) Western blotting determined Egfl7 levels in cell lysate and conditioned media from LFs or CAFs. (B) HPA database (https://www.proteinatlas.org/) showed that Egfl7 protein expression in normal hepatocytes and HCC tissues. (C) Egfl7 mRNA expression in normal tissues and tumor tissues in TIMER 2.0 database (http://timer.comp-genomics.org/). (D) The correlation between Egfl7 expression and α-SMA expression in HCC from TIMER 2.0 database. (E) The relationship between Egfl7 expression and CAF infiltration from TIMER 2.0 EPIC, MCPCOUNTER, XCELL, TIDE algorithms in HCC. (F) Western blotting determined Egfl7 expression levels in SLHCC and NHCC samples.


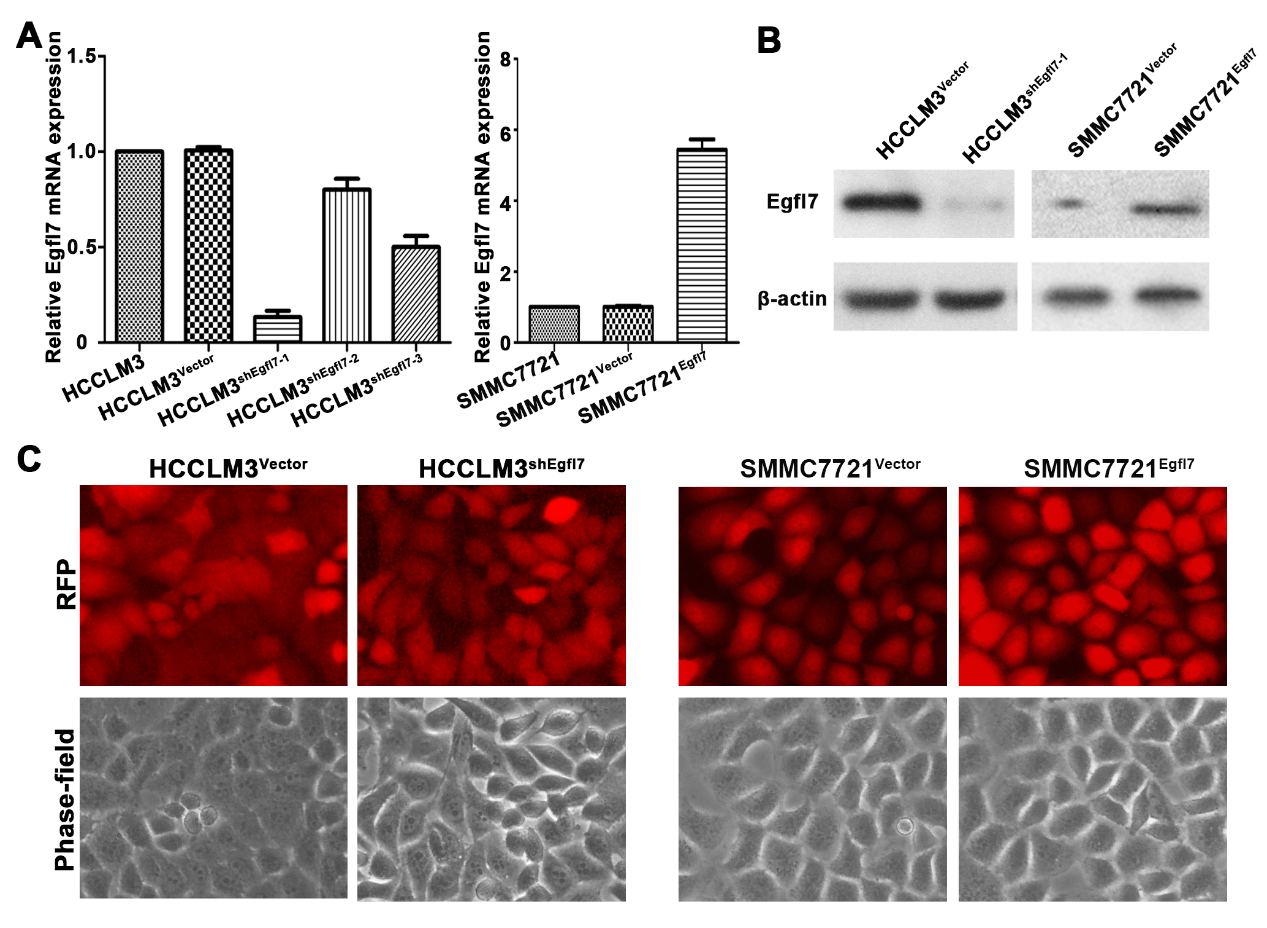


**Fig S5. Establishment of working cell lines.** (A-B) qRT-PCR (A) and western blotting (B) confirmed the efficiency of knockdown and overexpression of Egfl7 in indicated HCC cell lines. (C) Successfully transfecting indicated HCC cells with RFP gene.


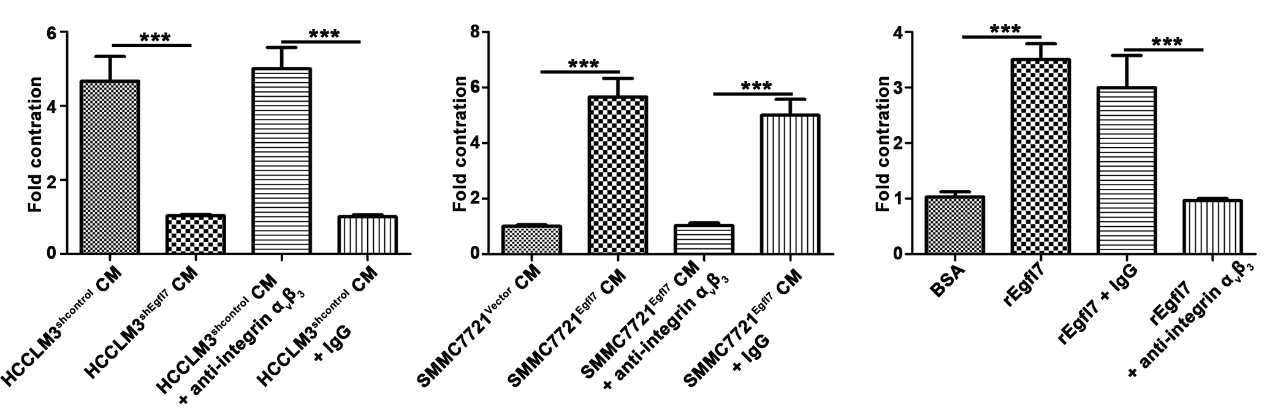


**Fig S6.** Collagen gel contraction assays were used to detect contraction ability of liver fibroblasts (LFs) cocultured with HCCLM3^shEgfl7^, SMMC7721^Egfl7^, or their control cells, or recombinant Egfl7 protein (rEgfl7) after adding or not adding α_ν_β_3_ integrin neutralizing antibody. ****P* < 0.001.


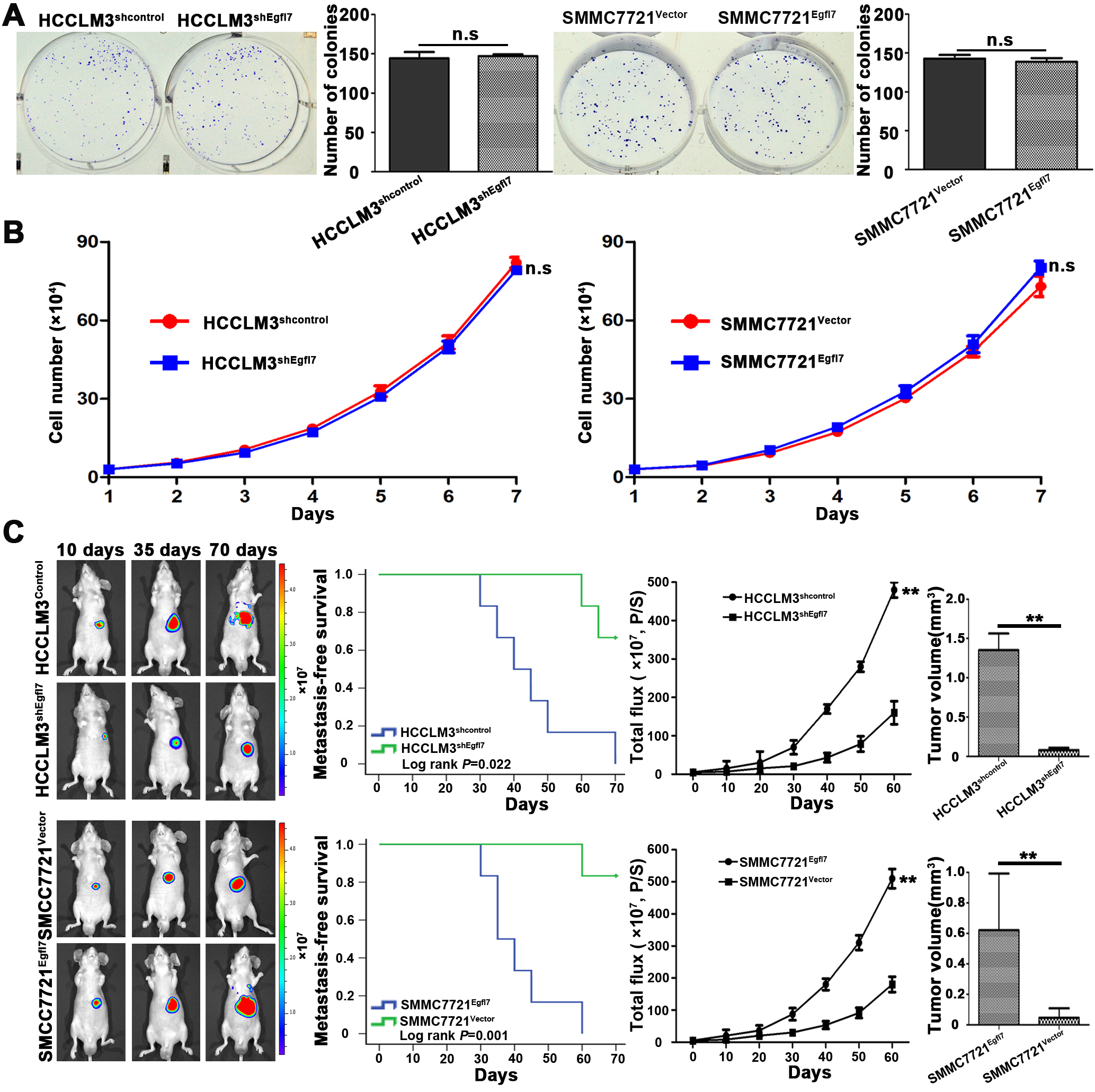


**Fig S7. *In vitro* and *in vivo* assays to confirm the biological roles of Egfl7 in HCC cells.** (A-B) Colony formation assays (A) and MTT assays (B) were used to determine the proliferative ability of HCC cells after knocking down or overexpressing Egfl7. (C) Orthotopic tumors of mouse were constructed using HCCLM3^shEgfl7^, SMMC-7721^Egfl7^ and their control cells. The mice survival and growth curves were depicted and the final tumor volumes were compared. n.s, no significance; ***P* < 0.01.
