# Supplementary material for: Hepatocellular carcinoma cells remodel the pro-metastatic tumour microenvironment through recruitment and activation of fibroblasts via paracrine Egfl7 signaling
Source: Cell Commun Signal. 2023 Jul 21;21:180. doi: 10.1186/s12964-023-01200-6 (PMC10362567; doi:10.1186/s12964-023-01200-6)
Supplement: Supplementary file 3 — Additional file 2: Table S1. Clinicopathologic variables of HCC patients in the training cohort and validation cohort. Table S2. The primers of gene used for qRT-PCR. Table S3. Antibodies used in this study. Table S4. The Cox proportional hazard regression analyses for disease-free survival (DFS) and overall survival (OS) in the training cohort. Table S5. The Cox proportional hazard regression analyses for disease-free survival (DFS) and overall survival (OS) in the validation cohort. Table S6. A list of the secreted proteins differentially expressed by HCC cells with different metastatic potential. [file 12964_2023_1200_MOESM2_ESM.doc]

**Table S1. Clinicopathologic Variables of HCC Patients in the Training Cohort and Validation Cohort**

| **Clinicopathologic Variables** | **Counts** | | ***P* value** |
| --- | --- | --- | --- |
|  | **Training cohort (n=176)** | **Validation cohort (n=99)** |  |
| **Gender** |  |  |  |
| Female | 30 | 24 | 0.149 |
| Male | 146 | 75 |  |
| **Age (years)** |  |  |  |
| <60 | 133 | 84 | 0.070 |
| ≥60 | 43 | 15 |  |
| **AFP (ng/mL)** |  |  |  |
| ≤ 20 | 65 | 31 | 0.348 |
| > 20 | 111 | 68 |  |
| **HBsAg** |  |  |  |
| Negative | 54 | 23 | 0.187 |
| Positive | 122 | 76 |  |
| **Liver cirrhosis** |  |  |  |
| Absence | 73 | 43 | 0.752 |
| Presence | 103 | 56 |  |
| **Child-Pugh classification** |  |  |  |
| A | 155 | 89 | 0.645 |
| B | 21 | 10 |  |
| **Tumor number** |  |  |  |
| Solitary | 69 | 40 | 0.845 |
| Multiple (≥ 2) | 107 | 59 |  |
| **Tumor size (cm)** |  |  |  |
| ≤ 5 | 61 | 41 | 0.266 |
| > 5 | 115 | 58 |  |
| **Microvascular invasion** |  |  |  |
| Absence | 76 | 39 | 0.541 |
| Presence | 100 | 60 |  |
| **Capsular formation** |  |  |  |
| Absence | 103 | 67 | 0.296 |
| Presence | 73 | 32 |  |
| **Edmondson-Steiner grade** |  |  |  |
| I-II | 80 | 49 | 0.462 |
| III-IV | 96 | 50 |  |
| **TNM stage** |  |  |  |
| I | 55 | 37 | 0.302 |
| II-III | 121 | 62 |  |
| **BCLC stage** |  |  |  |
| 0-A | 52 | 34 | 0.410 |
| B-C | 124 | 65 |  |

**Table S2. The primers of gene used for qRT-PCR**

| **Common synonyms** | **Full names** | **GenBank Accession** | **Forward primers** | **Reverse primers** |
| --- | --- | --- | --- | --- |
| Eglf7 | Epidermal growth factor (EGF)-like domain 7 | [NM_016215.4](http://www.ncbi.nlm.nih.gov/nuccore/NM_016215.4) | TGATGTGGCTTCTGGTGTTG | AGATGGTTCGGTAGGTGCTG |
| VEGF | Vascular endothelial growth factor | [NM_001025366.2](http://www.ncbi.nlm.nih.gov/nuccore/NM_001025366.2) | GTCCAACTTCTGGGCTGTCT | CCCCCTCTCCTCTTCTTCTC |
| TGFB1 | Transforming growth factor, beta-induced, 68kDa | [NM_000660.5](http://www.ncbi.nlm.nih.gov/nuccore/NM_000660.5) | CTGGCGATACCTCAGCAAC | TAAGGCGAAAGCCCTCAAT |
| POSTN | Periostin, osteoblast specific factor | [NM_001135934.1](http://www.ncbi.nlm.nih.gov/nuccore/NM_001135934.1) | TCCTGATTCTGCCAAACAAGT | GCCAGCAAAGTGTATTCTCCA |
| HGF | Hepatic growth factor | [NM_000601.4](http://www.ncbi.nlm.nih.gov/nuccore/NM_000601.4) | AGCAAGAAAACAATGCCTCTG | TGCGTCCTTTACCAATGATG |
| BMP1 | Bone morphogenetic protein 1 | [NM_001199.3](http://www.ncbi.nlm.nih.gov/nuccore/NM_001199.3) | CCCCAAGTATGAGGTGAACG | GGAGAAGTTGCCTGTGCTGT |
| IGF2 | Insulin-like growth factor 2 | [NM_000612.5](http://www.ncbi.nlm.nih.gov/nuccore/NM_000612.5) | ATCGTTGAGGAGTGCTGTTTC | GGGGTATCTGGGGAAGTTGT |
| G-CSF | Granulocyte-colony stimulating factor | [NM_000759.3](http://www.ncbi.nlm.nih.gov/nuccore/NM_000759.3) | TGCTGCTCGGACACTCTCT | AGCCCCTGGTAGAGGAAAAG |
| GM-CSF | GM-colony stimulating factor | [NM_000758.3](http://www.ncbi.nlm.nih.gov/nuccore/NM_000758.3) | CAGCCACTACAAGCAGCAC | GGGGATGACAAGCAGAAAGT |
| OPN | Osteopontin | [NM_000582.2](http://www.ncbi.nlm.nih.gov/nuccore/NM_000582.2) | GTGATTTGCTTTTGCCTCCT | GAGATGGGTCAGGGTTTAGC |
| PDGF-D | platelet-derived growth factor D | [NM_025208.4](http://www.ncbi.nlm.nih.gov/nuccore/NM_025208.4) | GATGATGCCAAGCGTTACAG | GATGATGCCAAGCGTTACAG |
| NOTCH | Notch Receptor 1 | NM_017617.5 | GAGGCGTGGCAGACTATGC | CTTGTACTCCGTCAGCGTGA |
| EGFR | Epidermal Growth Factor Receptor | NM_201284.2 | TTGCCGCAAAGTGTGTAACG | GTCACCCCTAAATGCCACCG |
| ITGAV | Integrin Subunit Alpha V | NM_001145000 | ATCTGTGAGGTCGAAACAGGA | TGGAGCATACTCAACAGTCTTTG |
| ITGB3 | Integrin Subunit Beta 3 | NM_000212 | GTGACCTGAAGGAGAATCTGC | CCGGAGTGCAATCCTCTGG |
| GAPDH | glyceraldehyde-3-phosphate dehydrogenase | NM_001256799.2 | GCACCGTCAAGGCTGAGAAC | TGGTGAAGACGCCAGTGGA |

Table S3. Antibodies used in this study

| Names | Sources | Species | Working dilution | | | | |
| --- | --- | --- | --- | --- | --- | --- | --- |
|  |  |  | WB |  | IHC |  | IF |
| β-actin | Sigma Aldrich | Mouse | 1:1000 |  | - |  | - |
| α-SMA | Abcam | Mouse | 1:400 |  | 1:200 |  | 1:100 |
| Egfl7 | Santa Cruz | Rabbit | 1:400 |  | 1:200 |  | - |
| p-ERK1/2 | Santa Cruz | Mouse | 1:1000 |  | - |  | - |
| ERK1/2 | Santa Cruz | Mouse | 1:1000 |  | - |  | - |
| p-FAK | Cell Signaling Technology | Mouse | 1:1000 |  | - |  | - |
| FAK | Cell Signaling Technology | Mouse | 1:1000 |  | - |  | - |
| p-AKT | Cell Signaling Technology | Mouse | 1:500 |  | - |  | - |
| AKT | Cell Signaling Technology | Mouse | 1:500 |  | - |  | - |
| Vimentin | Santa Cruz | Mouse | 1:500 |  | - |  | 1:100 |
| CD34 | Santa Cruz | Mouse | 1:1000 |  | - |  | 1:200 |
| CD45 | Santa Cruz | Mouse | 1:1000 |  | - |  | 1:200 |
| pan-CKs | Biolegend | Mouse | 1:1000 |  | - |  | 1:100 |
| RFP | Abcam | Rabbit | - |  | - |  | 1:100 |

**Table S4. The Cox Proportional Hazard Regression Analyses for Disease-free Survival (DFS) and Overall Survival (OS) in the Training C**ohort

| **Variables** | **No.** | **DFS** | | | | |  | **OS** | | | | |
| --- | --- | --- | --- | --- | --- | --- | --- | --- | --- | --- | --- | --- |
|  |  | **Univariable Analysis** | |  | **Multivariable Analysis** | |  | **Univariable Analysis** | |  | **Multivariable Analysis** | |
|  |  | **HR (95% CI)** | ***P* value** |  | **HR (95% CI)** | ***P* value** |  | **HR (95% CI)** | ***P* value** |  | **HR (95% CI)** | ***P* value** |
| **Gender** |  |  |  |  |  |  |  |  |  |  |  |  |
| Female | 30 | Reference |  |  |  |  |  | Reference |  |  |  |  |
| Male | 146 | 1.076(0.598-1.936) | 0.309 |  |  | NA |  | 1.034(0.526-2.032) | 0.214 |  |  | NA |
| **Age (years)** |  |  |  |  |  |  |  |  |  |  |  |  |
| ≤60 | 133 | Reference |  |  |  |  |  | Reference |  |  |  |  |
| >60 | 43 | 1.285(0.826-1.982) | 0.121 |  |  | NA |  | 1.314(0.726-2.371) | 0.109 |  |  | NA |
| **AFP (ng/mL)** |  |  |  |  |  |  |  |  |  |  |  |  |
| ≤ 20 | 65 | Reference |  |  | Reference |  |  | Reference |  |  |  |  |
| > 20 | 111 | 1.527(0.906-2.574) | 0.073 |  | 1.106(0.712-1.718) | 0.136 |  | 1.302(0.704-2.408) | 0.178 |  |  | NA |
| **Hepatitis B status** |  |  |  |  |  |  |  |  |  |  |  |  |
| Negative | 54 | Reference |  |  |  |  |  | Reference |  |  |  |  |
| Positive | 122 | 1.328(0.694-2.541) | 0.203 |  |  | NA |  | 1.137(0.903-1.432) | 0.304 |  |  | NA |
| **Liver cirrhosis** |  |  |  |  |  |  |  |  |  |  |  |  |
| Absence | 73 | Reference |  |  |  |  |  | Reference |  |  | Reference |  |
| Presence | 103 | 1.217(0.712-2.080) | 0.252 |  |  | NA |  | 1.349(1.072-1.698) | **0.044** |  | 1.109(0.803-1.532) | 0.141 |
| **Child-Pugh classification** |  |  |  |  |  |  |  |  |  |  |  |  |
| A | 155 | Reference |  |  |  |  |  | Reference |  |  |  |  |
| B | 21 | 1.097(0.624-1.928) | 0.289 |  |  | NA |  | 1.342(0.863-2.087) | 0.106 |  |  | NA |
| **Tumor number** |  |  |  |  |  |  |  |  |  |  |  |  |
| Solitary | 69 | Reference |  |  | Reference |  |  | Reference |  |  | Reference |  |
| Multiple | 107 | 3.024(1.941-4.712) | **0.008** |  | 2.019(1.613-2.527) | **0.036** |  | 2.704(1.625-4.497) | **0.010** |  | 1.937(1.429-2.626) | **0.041** |
| **Tumor size** |  |  |  |  |  |  |  |  |  |  |  |  |
| ≤5 cm | 61 | Reference |  |  |  |  |  | Reference |  |  | Reference |  |
| >5 cm | 115 | 1.365(0.769-2.423) | 0.253 |  |  | NA |  | 1.506(1.251-1.812) | **0.039** |  | 1.084(0.816-1.441) | 0.103 |
| **Capsular formation** |  |  |  |  |  |  |  |  |  |  |  |  |
| Absence | 103 | Reference |  |  | Reference |  |  | Reference |  |  |  |  |
| Presence | 73 | 0.634(0.426-0.904) | **0.041** |  | 0.836(0.658-1.302) | 0.103 |  | 0.895(0.637-1.346) | 0.113 |  |  | NA |
| **Microvascular invasion** |  |  |  |  |  |  |  |  |  |  |  |  |
| Absence | 76 | Reference |  |  | Reference |  |  | Reference |  |  | Reference |  |
| Presence | 100 | 4.057(2.485-6.623) | **<0.001** |  | 3.048(1.862-4.989) | **0.001** |  | 3.836(2.518-5.843) | **<0.001** |  | 2.705(1.746-4.191) | **0.007** |
| **Edmondson-Steiner grade** |  |  |  |  |  |  |  |  |  |  |  |  |
| Low grade (I and II) | 80 | Reference |  |  | Reference |  |  | Reference |  |  | Reference |  |
| High grade (III and IV) | 96 | 2.802(1.725-4.551) | **0.012** |  | 2.413(1.551-3.754) | **0.028** |  | 3.247(2.103-5.013) | **0.002** |  | 2.206(1.504-3.236) | **0.025** |
| **TNM Stage** |  |  |  |  |  |  |  |  |  |  |  |  |
| I | 55 | Reference |  |  | Reference |  |  | Reference |  |  | Reference |  |
| II- III | 121 | 3.854(2.009-7.393) | **0.002** |  | 2.524(1.715-3.715) | **0.013** |  | 3.634(2.327-5.675) | **0.006** |  | 2.801(1.503-5.219) | **0.023** |
| **BCLC Stage** |  |  |  |  |  |  |  |  |  |  |  |  |
| 0-A | 52 | Reference |  |  | Reference |  |  | Reference |  |  | Reference |  |
| B-C | 124 | 2.173(1.394-3.082) | **0.018** |  | 1.496(1.136-2.291) | **0.048** |  | 2.532(2.043-4.003) | **0.015** |  | 2.072(1.831-3.923) | **0.032** |
| **α-SMA expression** |  |  |  |  |  |  |  |  |  |  |  |  |
| Low | 70 | Reference |  |  | Reference |  |  | Reference |  |  | Reference |  |
| High | 106 | 5.854(3.193-10.733) | **<0.001** |  | 4.309(2.908-6.385) | **0.006** |  | 4.914(3.009-8.025) | **0.001** |  | 4.204(2.841-6.221) | **0.011** |

**Table S5. The Cox Proportional Hazard Regression Analyses for Disease-free Survival (DFS) and Overall Survival (OS) in the Validation C**ohort

| **Variables** | **No.** | **DFS** | | | | |  | **OS** | | | | |
| --- | --- | --- | --- | --- | --- | --- | --- | --- | --- | --- | --- | --- |
|  |  | **Univariable Analysis** | |  | **Multivariable Analysis** | |  | **Univariable Analysis** | |  | **Multivariable Analysis** | |
|  |  | **HR (95% CI)** | ***P* Value** |  | **HR (95% CI)** | ***P* Value** |  | **HR (95% CI)** | ***P* Value** |  | **HR (95% CI)** | ***P* Value** |
| **Gender** |  |  |  |  |  |  |  |  |  |  |  |  |
| Female | 24 | Reference |  |  |  |  |  | Reference |  |  |  |  |
| Male | 75 | 1.072(0.618-1.859) | 0.157 |  |  | NA |  | 1.023(0.734-1.426) | 0.301 |  |  | NA |
| **Age (years)** |  |  |  |  |  |  |  |  |  |  |  |  |
| ≤60 | 84 | Reference |  |  |  |  |  | Reference |  |  |  |  |
| >60 | 15 | 1.019(0.751-1.357) | 0.197 |  |  | NA |  | 1.307(0.629-2.078) | 0.159 |  |  | NA |
| **AFP (ng/mL)** |  |  |  |  |  |  |  |  |  |  |  |  |
| ≤ 20 | 31 | Reference |  |  | 1 |  |  | Reference |  |  |  |  |
| > 20 | 68 | 1.603(1.108-2.319) | **0.042** |  | 1.180(0.904-1.540) | 0.146 |  | 1.216(0.835-1.771) | 0.198 |  |  | NA |
| **Hepatitis B status** |  |  |  |  |  |  |  |  |  |  |  |  |
| Negative | 23 | Reference |  |  |  |  |  | Reference |  |  |  |  |
| Positive | 76 | 1.058(0.785-1.426) | 0.182 |  |  | NA |  | 1.074(0.832-1.386) | 0.249 |  |  | NA |
| **Liver cirrhosis** |  |  |  |  |  |  |  |  |  |  |  |  |
| Absence | 43 | Reference |  |  |  |  |  | Reference |  |  | Reference |  |
| Presence | 56 | 1.207(0.620-2.349) | 0.145 |  |  | NA |  | 1.723(1.078-2.754) | **0.047** |  | 1.215(0.902-1.637) | 0.094 |
| **Child-Pugh classification** |  |  |  |  |  |  |  |  |  |  |  |  |
| A | 89 | Reference |  |  |  |  |  | Reference |  |  |  |  |
| B | 10 | 1.140(0.841-1.545) | 0.176 |  |  | NA |  | 1.134(0.792-1.624) | 0.209 |  |  | NA |
| **Tumor number** |  |  |  |  |  |  |  |  |  |  |  |  |
| Solitary | 40 | Reference |  |  | Reference |  |  | Reference |  |  | Reference |  |
| Multiple | 59 | 3.904(2.605-5.851) | **0.003** |  | 3.314(2.191-5.013) | **0.011** |  | 3.582(2.723-4.712) | **0.009** |  | 2.832(2.054-3.905) | **0.023** |
| **Tumor size** |  |  |  |  |  |  |  |  |  |  |  |  |
| ≤5 cm | 41 | Reference |  |  |  |  |  | Reference |  |  |  |  |
| >5 cm | 58 | 1.402(0.834-2.357) | 0.129 |  |  | NA |  | 1.372(0.883-2.132) | 0.151 |  |  | NA |
| **Capsular formation** |  |  |  |  |  |  |  |  |  |  |  |  |
| Absence | 67 | Reference |  |  |  |  |  | Reference |  |  | Reference |  |
| Presence | 32 | 1.505(0.641-3.534) | 0.106 |  |  | NA |  | 1.685(1.017-2.792) | **0.048** |  | 1.038(0.957-1.126) | 0.109 |
| **Microvascular invasion** |  |  |  |  |  |  |  |  |  |  |  |  |
| Absence | 39 | Reference |  |  | Reference |  |  | Reference |  |  | Reference |  |
| Presence | 60 | 4.725(3.051-7.317) | **<0.001** |  | 3.128(2.536-3.858) | **0.013** |  | 4.982(3.087-8.040) | **<0.001** |  | 3.526(2.354-5.282) | **0.010** |
| **Edmondson-Steiner grade** |  |  |  |  |  |  |  |  |  |  |  |  |
| Low grade (I and II) | 49 | Reference |  |  | Reference |  |  | Reference |  |  | Reference |  |
| High grade (III and IV) | 50 | 2.105(1.304-3.398) | **0.015** |  | 1.974(1.137-3.427) | **0.029** |  | 2.512(1.305-4.835) | **0.013** |  | 2.027(1.214-3.384) | **0.035** |
| **TNM Stage** |  |  |  |  |  |  |  |  |  |  |  |  |
| I | 37 | Reference |  |  | Reference |  |  | Reference |  |  | Reference |  |
| II- III | 62 | 4.061(2.708-6.090) | **0.005** |  | 3.214(1.975-5.230) | **0.016** |  | 4.309(2.834-6.552) | **0.006** |  | 3.073(2.098-4.501) | **0.019** |
| **BCLC Stage** |  |  |  |  |  |  |  |  |  |  |  |  |
| 0-A | 34 | Reference |  |  | Reference |  |  | Reference |  |  | Reference |  |
| B-C | 65 | 2.896(1.658-5.058) | **0.007** |  | 1.857(1.325-2.603) | **0.031** |  | 2.086(1.372-3.173) | **0.023** |  | 1.843(1.452-2.339) | **0.044** |
| **α-SMA expression** |  |  |  |  |  |  |  |  |  |  |  |  |
| Low | 42 | Reference |  |  | Reference |  |  | Reference |  |  | Reference |  |
| High | 57 | 3.943(2.561-6.071） | **0.009** |  | 2.743(1.856-4.053) | **0.022** |  | 3.802(1.823-7.928) | **0.017** |  | 2.463(1.536-3.942) | **0.031** |

**Table S6. A list of the secreted proteins differentially expressed by HCC cells with different metastatic potential. The data were obtained through qRT-PCR analysis of mRNA expression of a series of secreted proteins in HCC cells with different metastatic potential. Relative mRNA expression levels were calculated by the 2^-∆Ct^** method based on the threshold cycle (Ct) values and were normalized to the internal control of GAPDH

|  |  | **the expression of Egfl7 mRNA in low metastatic HCC cells (**2^-∆Ct^**)** | | |  | **the expression of Egfl7 mRNA in high metastatic HCC cells (**2^-∆Ct^**)** | | | ***P* value**  **(high *vs.* low)** |
| --- | --- | --- | --- | --- | --- | --- | --- | --- | --- |
|  |  | SMMC-7721 | Bel-7402 | HepG2 |  | Hep3B | MHCC97-H | HCCLM3 |  |
| Eglf7 | epidermal growth factor (EGF)-like domain 7 | 0.0015 | 0.0013 | 0.0151 |  | 0.0736 | 0.0896 | 0.1467 | **0.012** |
| VEGF | Vascular endothelial growth factor | 0.0110 | 0.0132 | 0.0091 |  | 0.0102 | 0.0083 | 0.0124 | 0.657 |
| TGFB1 | Transforming growth factor, beta-induced, 68kDa | 0.0113 | 0.0081 | 0.0052 |  | 0.0034 | 0.0195 | 0.0272 | 0.304 |
| POSTN | Periostin, osteoblast specific factor | 0.0012 | 0.0008 | 0.0025 |  | 0.0033 | 0.0008 | 0.0014 | 0.702 |
| HGF | Hepatic growth factor | 0.0039 | 0.0028 | 0.0045 |  | 0.0042 | 0.0037 | 0.0051 | 0.404 |
| BMP1 | Bone morphogenetic protein 1 | 0.0014 | 0.0018 | 0.0020 |  | 0.0031 | 0.0017 | 0.0036 | 0.101 |
| IGF2 | Insulin-like growth factor 2 | 0.0072 | 0.0004 | 0.0015 |  | 0.0021 | 0.0019 | 0.0032 | 0.782 |
| G-CSF | Granulocyte-colony stimulating factor | 0.0017 | 0.0006 | 0.0023 |  | 0.0035 | 0.007 | 0.0011 | 0.261 |
| GM-CSF | Granulocyte-macrophage colony-stimulating factor | 0.0001 | 0.0003 | 0.0004 |  | 0.0002 | 0.0006 | 0.0010 | 0.248 |
| OPN | Osteopontin | 0.0018 | 0.0003 | 0.0022 |  | 0.0002 | 0.0016 | 0.0035 | 0.757 |
| PDGF-D | platelet-derived growth factor D | 0.0001 | 0.0004 | 0.0001 |  | 0.0003 | 0.0006 | 0.0013 | 0.163 |
